# Supplementary material for: Structural asymmetry governs the assembly and GTPase activity of McrBC restriction complexes
Source: Nat Commun. 2020 Nov 20;11:5907. doi: 10.1038/s41467-020-19735-4 (PMC7680126; doi:10.1038/s41467-020-19735-4)
Supplement: Supplementary file 1 — Supplementary Information [file 41467_2020_19735_MOESM1_ESM.pdf]

# Supplementary Information for:

# Structural asymmetry governs the assembly and GTPase activity of McrBC restriction complexes

Yiming Niu[1,2,3,6], Hiroshi Suzuki[2,4,6], Christopher J. Hosford[1,5], Thomas Walz[2*], and Joshua S. Chappie[1*]

[1] Department of Molecular Medicine, Cornell University, Ithaca, NY, USA

[2] Laboratory of Molecular Electron Microscopy, The Rockefeller University, New York, NY, USA

[3] present address: Laboratory Molecular Neurobiology and Biophysics, The Rockefeller University, New York, NY, USA

[4] present address: Advanced Research Institute, Tokyo Medical and Dental University, Tokyo, Japan

[5] present address: New England Biolabs, Inc., Ipswich, MA, USA

[6] These authors contributed equally: Yiming Niu, Hiroshi Suzuki

* To whom correspondence should be addressed: twalz@rockefeller.edu (T.W.), chappie@cornell.edu (J.S.C.)

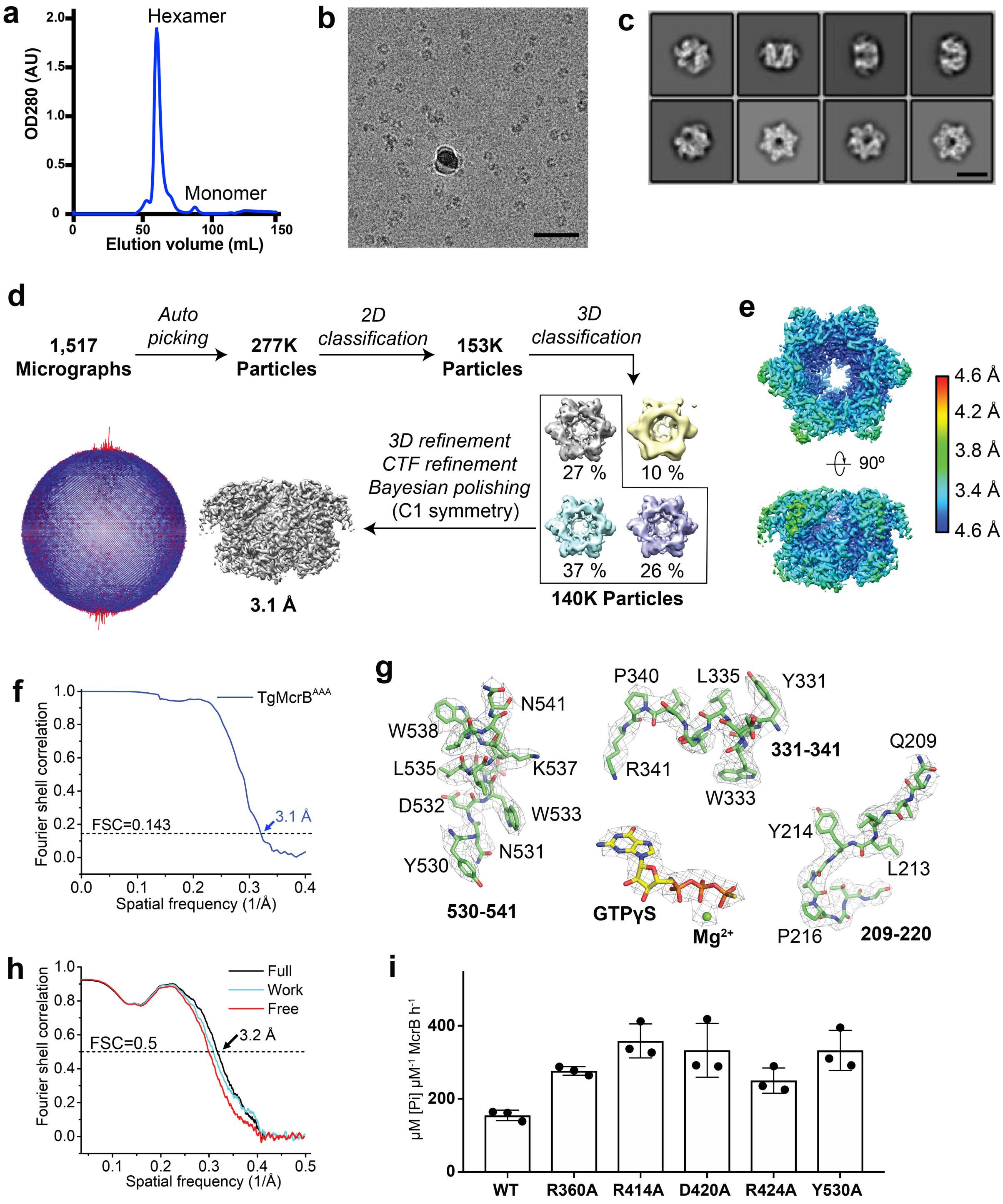
a
Hexamer
Monomer
OD280 (AU)
Elution volume (mL)
b
c
d
1,517 Micrographs
Auto picking
277K Particles
2D classification
153K Particles
3D classification
27 %
10 %
37 %
26 %
140K Particles
3D refinement
CTF refinement
Bayesian polishing
(C1 symmetry)
3.1 Å
e
90º
4.6 Å
4.2 Å
3.8 Å
3.4 Å
4.6 Å
f
TgMcrB^AAA
FSC=0.143
3.1 Å
Fourier shell correlation
Spatial frequency (1/Å)
g
N541
W538
L535
K537
D532
W533
Y530
N531
530-541
P340
L335
Y331
R341
331-341
W333
Q209
Y214
L213
GTPγS
Mg²⁺
P216
209-220
h
Full
Work
Free
FSC=0.5
3.2 Å
Fourier shell correlation
Spatial frequency (1/Å)
i
μM [Pi] μM⁻¹ McrB h⁻¹
WT
R360A
R414A
D420A
R424A
Y530A

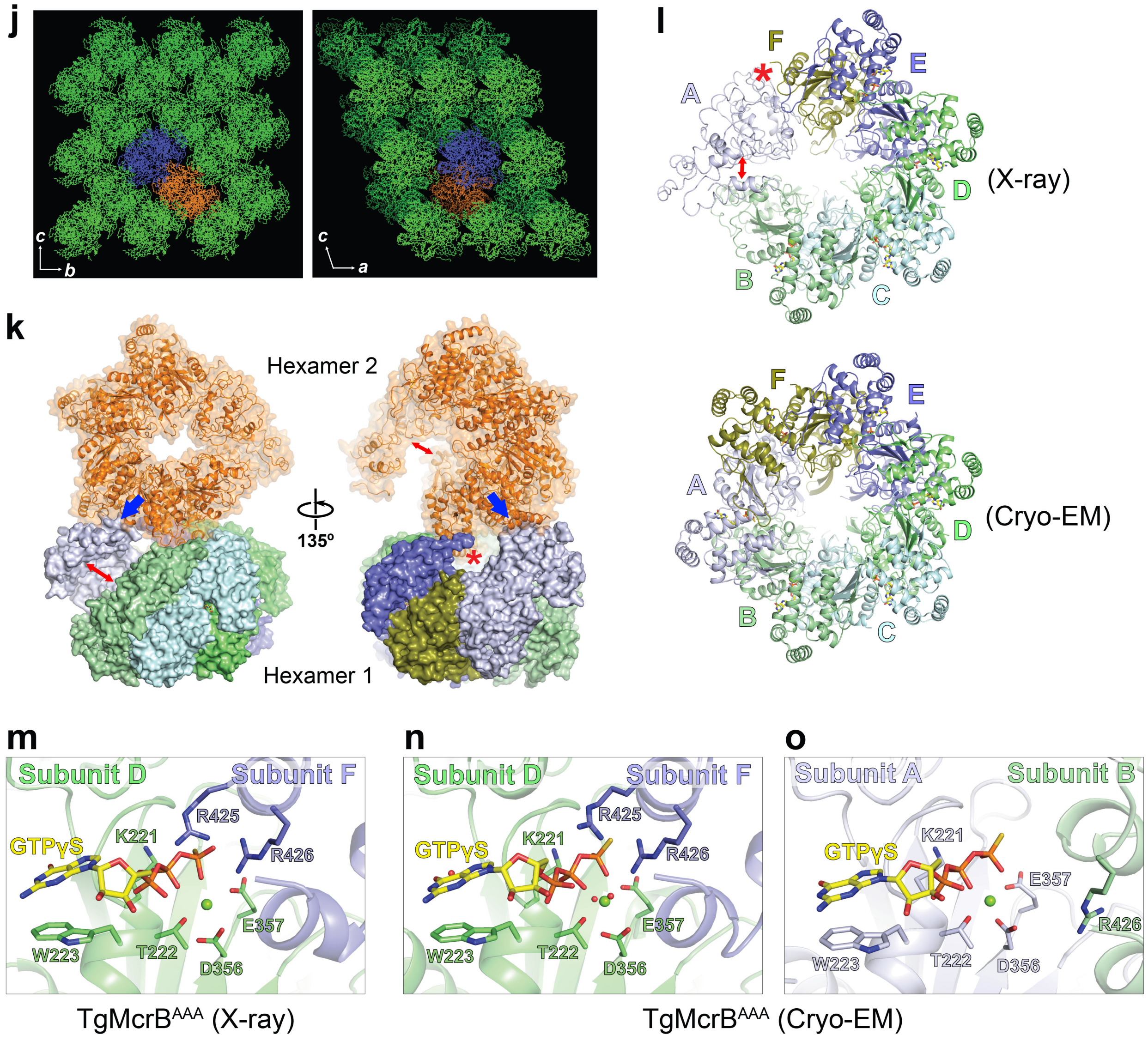


**Supplementary Figure 1: Biochemical and structural analysis of the TgMcrB$^{AAA}$ hexamer.** (**a**) Gel-filtration profile of the TgMcrB$^{AAA}$ hexamer in the absence of nucleotides. (**b**) Cryo-EM image area of vitrified TgMcrB$^{AAA}$ hexamers in the presence of 2.5 mM GTPγS. Scale bar is 50 nm. (**c**) Selected 2D-class averages of the TgMcrB$^{AAA}$ hexamer. Scale bar is 10 nm. (**d**) Cryo-EM data-processing workflow for the TgMcrB$^{AAA}$ hexamer and angular distribution plot of all TgMcrB$^{AAA}$ particles that contributed to the final map. The map and the angular distribution plot are shown in the same orientation. (**e**) Local resolution map for the TgMcrB$^{AAA}$ hexamer. (**f**) Gold-standard Fourier shell correlation (FSC) curve for the TgMcrB$^{AAA}$ hexamer after correction for masking effects. The resolution was estimated based on the FSC = 0.143 criterion. (**g**) Cryo-EM densities for selected regions in the map of the TgMcrB$^{AAA}$ hexamer. (**h**) Cross-validation FSC curves for the TgMcrB$^{AAA}$ hexamer: cyan curve, refined model *versus* half map 1 used for

refinement (Work); red curve, refined model *versus* half map 2 not used for refinement (Free); black curve, refined model *versus* the combined final map (Full). The similarity of the 'work' and 'free' curves suggests no substantial over-fitting. The correlation is above 0.5 up to a resolution of 3.2 Å. (**i**) GTPase activity of wild-type $TgMcrB^{AAA}$ and mutants in which alanine substitutions were introduced at residues at the tight and loose interfaces shown in Figure 1d and e (n = 3, mean ± standard deviation). (**j**) Crystal packing of $TgMcrB^{AAA}$ in the $P2_1$ space group, viewed perpendicular to the *b*-*c* plane (left panel) and the *a*-*c* plane (right panel). One crystallographically-unique molecule (hexamer 1) is shown in blue along with a symmetry-related molecule (hexamer 2) colored orange. Additional molecules within the lattice are colored green. (**k**) Two views of the 'open-ring' assembly of the $TgMcrB^{AAA}$ hexamer determined by X-ray crystallography. The subunits in hexamer 1 are colored as in Figure 1, and symmetry-related hexamer 2 is colored in orange. The red double arrow indicates the subunit separation at the A/B interface likely introduced by the crystal packing, while the red asterisk indicates the position of the small domain of subunit F, which was disordered and not seen in the crystal structure. The blue arrows indicate the crystal contact occurring between subunit A in hexamer 1 and subunit C in hexamer 2. (**l**) Top-view comparison between the structures of the $TgMcrB^{AAA}$ hexamers determined by X-ray crystallography (upper panel) and cryo-EM (lower panel). The subunits are colored as in Figure 1. The red double arrow and asterisk indicate the subunit separation and the disordered region of subunit F, respectively. (**m**) Close-up view of the GTP-binding sites at the tight D/E interfaces in the X-ray structure of the $TgMcrB^{AAA}$ hexamer. (**n** and **o**) Close-up view of the GTP-binding sites at the tight D/E interfaces (**n**) and the loose A/B interface (**o**) in the cryo-EM structure of the $TgMcrB^{AAA}$ hexamer.

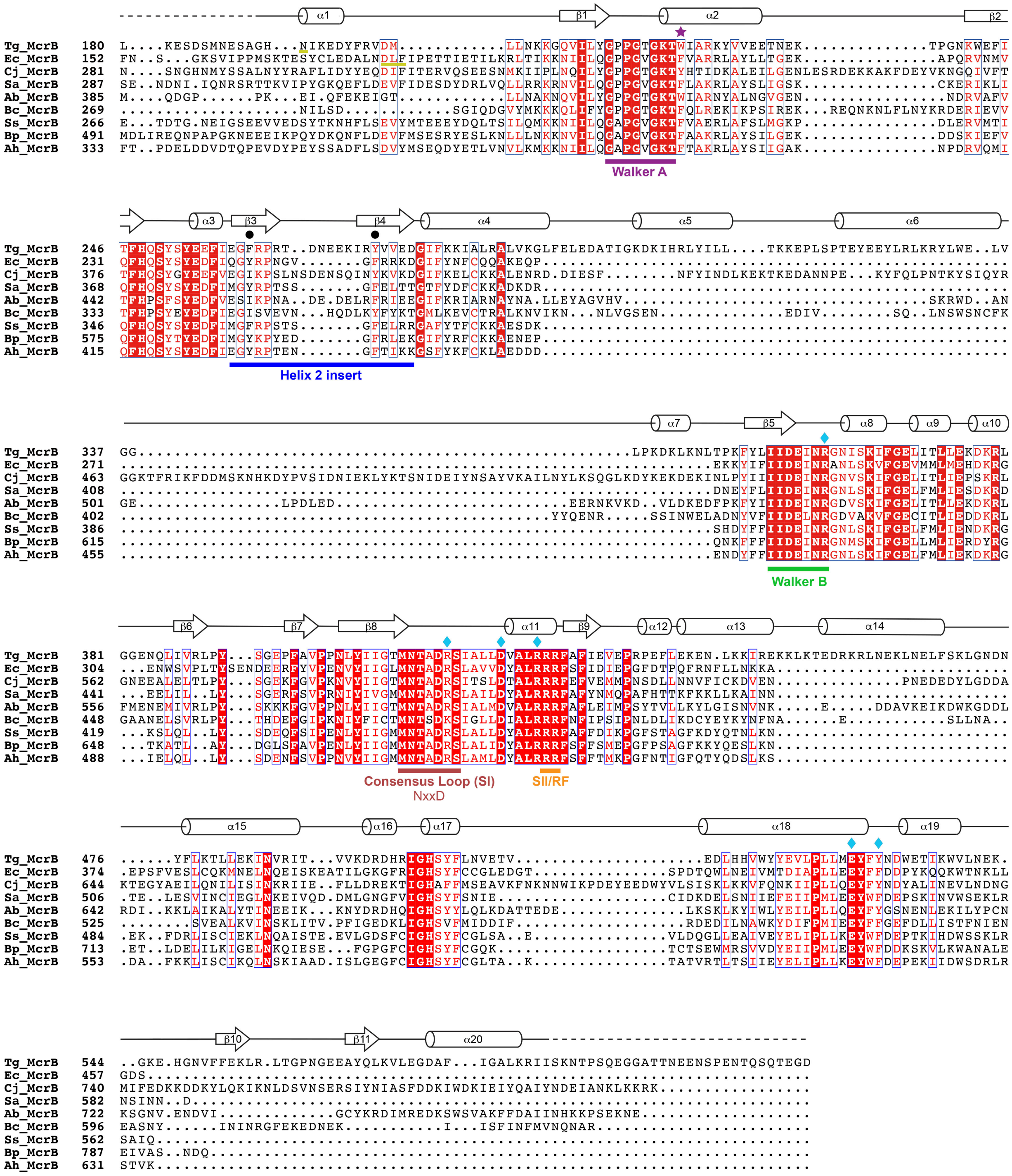


**Supplementary Figure 2: Sequence alignment of the AAA domains from McrB family proteins.** Conserved motifs are labeled and colored. The secondary structure diagram is based on the AAA domain of TgMcrB. Symbols denote the following: cyan diamonds, interface residues contributing to tight/loose asymmetry; black circles, hydrophobic residues that stabilize the loop-helix-loop region of the McrC finger domain; purple star, $\pi$-stacking residue that contacts the

guanine base. Segments near α1 involved in guanine nucleotide recognition are underlined in gold in Tg and EcMcrB sequences. Sequence shading indicates conservation: white text on red background, 100% conserved; boxed red text on white background, 70% conserved. Abbreviations are as follows: Tg, *Thermococcus gammatolerans*; Ec, *Escherichia coli*; Cj, *Campylobacter jejuni*; Sa, *Staphylococcus aureus*; Ab, *Aciduliprofundum boonei*; Bc, *Bacillus cereus*; Ss, *Streptococcus suis*; Bp, *Butyrivibrio proteoclasticus*; Ah, *Anaerobutyricum hallii*. The N-terminal DNA-binding domains are not conserved and therefore not shown here. The TgMcrB and EcMcrB sequences are 20% identical and 32% similar across the whole protein and 24% identical and 35% similar across the AAA+ domain.

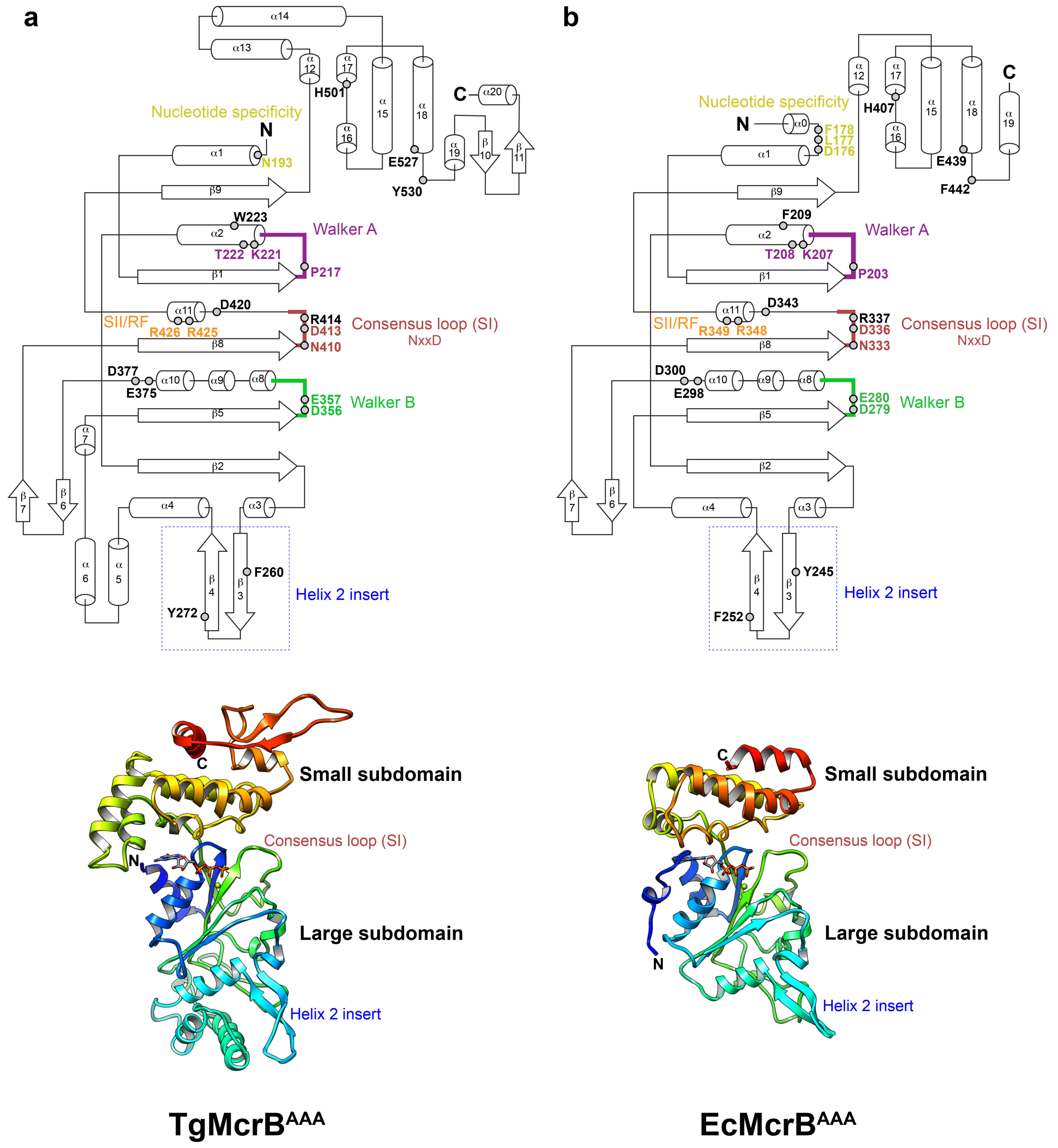


**Supplementary Figure 3: Secondary structure topology diagram and structures of the monomeric AAA+ domains of TgMcrB (a) and EcMcrB (b) in ribbon representation.** Conserved motifs among McrB family proteins are labeled and colored as in Supplementary Figure 2.

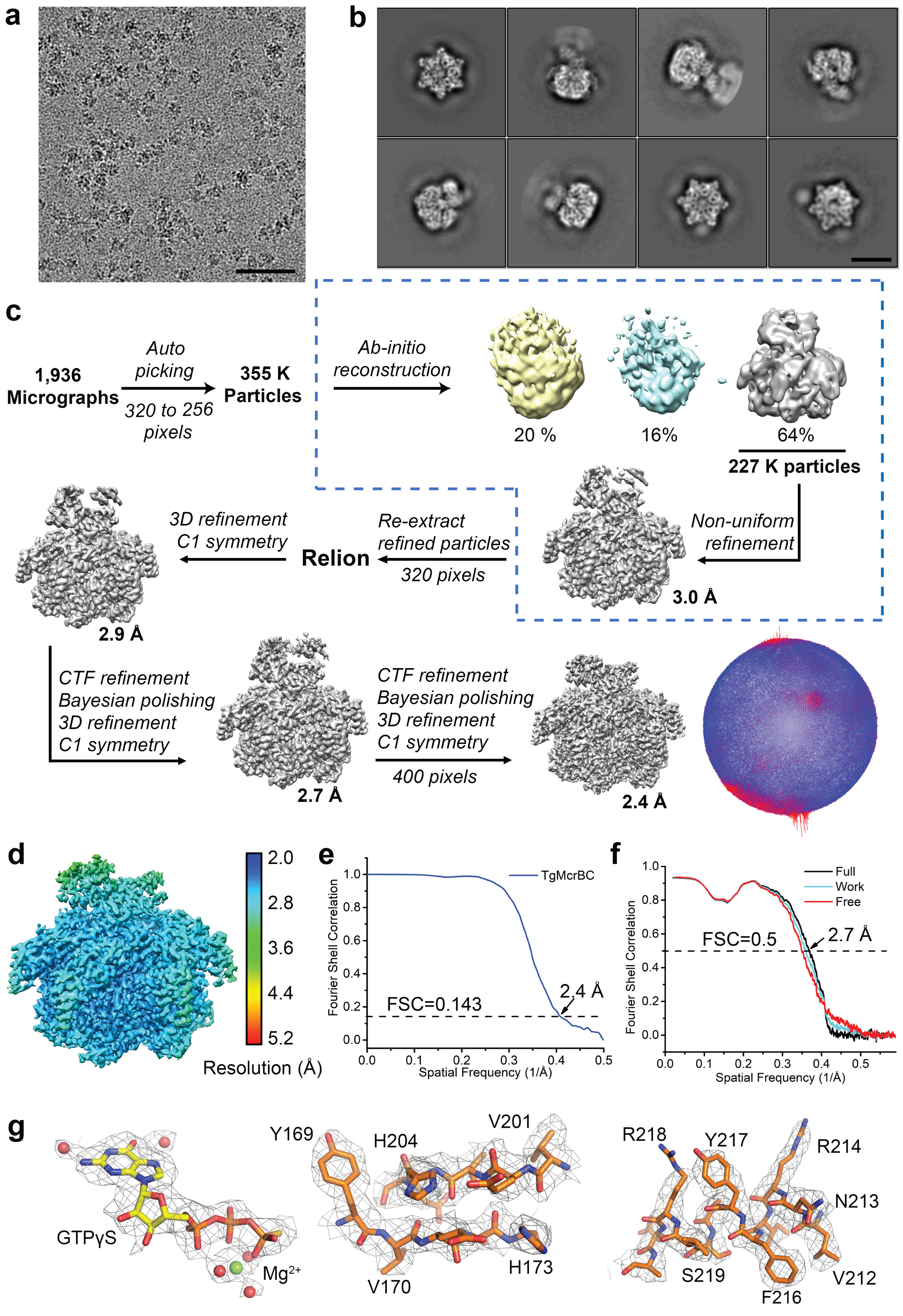

a
b
c
1,936 Micrographs
Auto picking
320 to 256 pixels
355 K Particles
Ab-initio reconstruction
20 %
16%
64%
227 K particles
Non-uniform refinement
3.0 Å
Re-extract refined particles
320 pixels
Relion
3D refinement C1 symmetry
2.9 Å
CTF refinement
Bayesian polishing
3D refinement
C1 symmetry
2.7 Å
CTF refinement
Bayesian polishing
3D refinement
C1 symmetry
400 pixels
2.4 Å
d
2.0
2.8
3.6
4.4
5.2
Resolution (Å)
e
TgMcrBC
Fourier Shell Correlation
FSC=0.143
2.4 Å
Spatial Frequency (1/Å)
f
Full
Work
Free
FSC=0.5
2.7 Å
Fourier Shell Correlation
Spatial Frequency (1/Å)
g
GTPγS
Mg2+
Y169
H204
V201
V170
H173
R218
Y217
R214
N213
S219
F216
V212

**Supplementary Figure 4: Cryo-EM analysis of the TgMcrBC complex.** (**a**) Cryo-EM image area of vitrified TgMcrBC complexes. Scale bar is 50 nm. (**b**) Selected 2D-class averages of the TgMcrBC complex. Scale bar is 10 nm. (**c**) Cryo-EM data-processing workflow for the TgMcrBC complex and angular distribution plot of all TgMcrBC particles that contributed to the final map. The map and the angular distribution plot are shown in the same orientation. (**d**) Local resolution map for the TgMcrBC half-complex. (**e**) Gold-standard FSC curve for the TgMcrBC 'half'-complex after correction for masking effects. The resolution was estimated based on the FSC = 0.143 criterion. (**f**) Cross-validation FSC curves for the TgMcrBC half-complex: cyan curve, refined model *versus* half map 1 used for refinement (Work); red curve, refined model *versus* half map 2 not used for refinement (Free); black curve, refined model *versus* the combined final map (Full). The similarity of the 'work' and 'free' curves suggests no substantial over-fitting. The correlation is above 0.5 up to a resolution of 2.7 Å. (**g**) Cryo-EM densities for selected regions of TgMcrB (left panel) and TgMcrC (middle and right panels) in the map of the TgMcrBC complex.

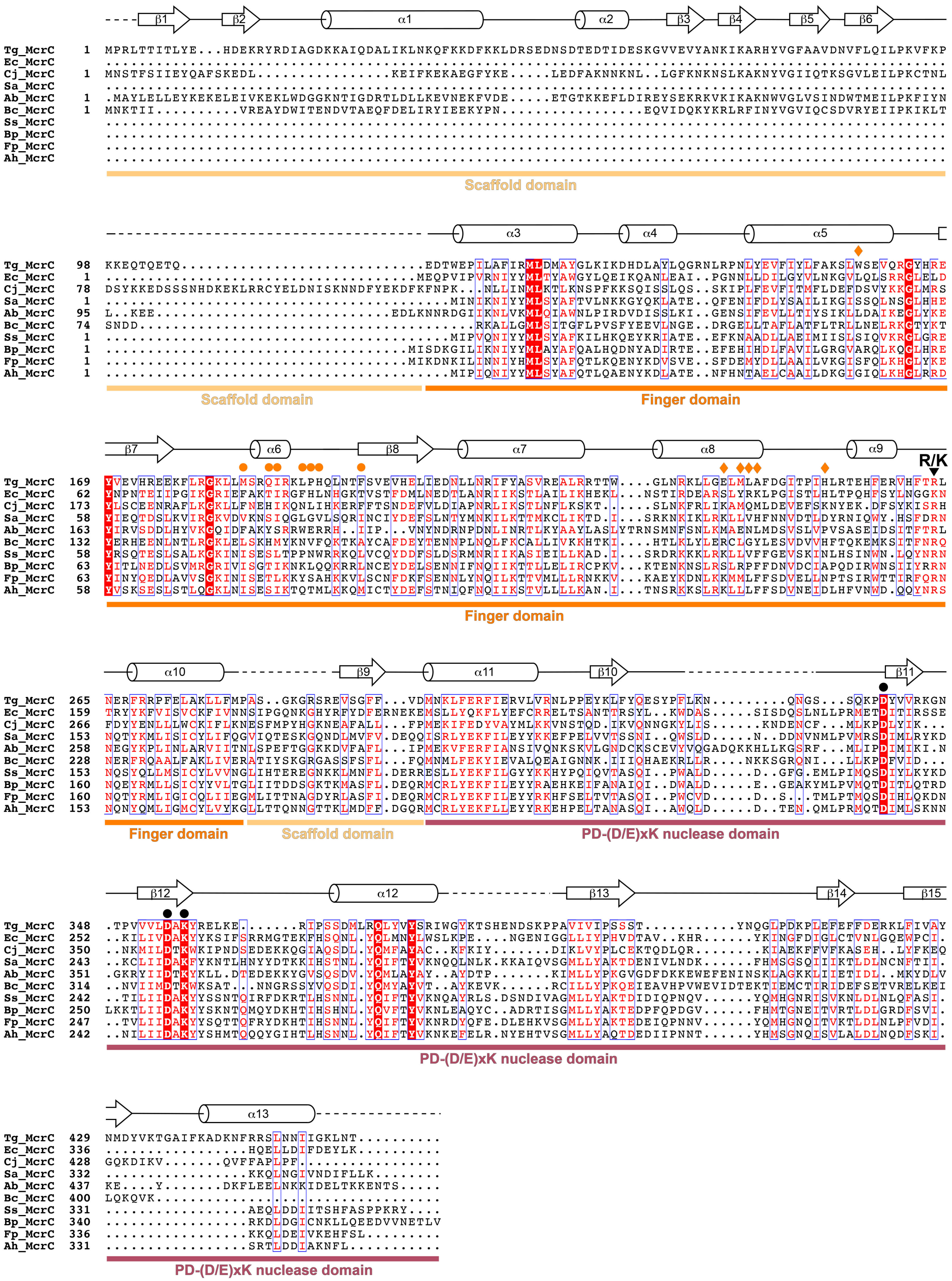
β1 β2 α1 α2 β3 β4 β5 β6
Tg_McrC
Ec_McrC
Cj_McrC
Sa_McrC
Ab_McrC
Bc_McrC
Ss_McrC
Bp_McrC
Fp_McrC
Ah_McrC
Scaffold domain
α3 α4 α5
Scaffold domain
Finger domain
β7 α6 β8 α7 α8 α9
R/K
Finger domain
α10 β9 α11 β10 β11
Finger domain
Scaffold domain
PD-(D/E)xK nuclease domain
β12 α12 β13 β14 β15
PD-(D/E)xK nuclease domain
α13
PD-(D/E)xK nuclease domain

**Supplementary Figure 5: Sequence alignment of McrC family proteins.** The secondary structure diagram is based on TgMcrC. Symbols denote the following: orange circle, loop-helix-loop residues interacting with hydrophobic side chains at the base of the McrB ring (Figure 3e); orange diamonds, residues that form anchoring interactions at the distorted tight McrB interface (Figure 3f); black triangle, conserved arginine or lysine whose side chain is important for the stimulation of McrB GTPase activity; black circles, residues critical for McrC nuclease activity. Individual domain segments are colored as in Figure 3d and labeled below the alignment. Sequence shading indicates conservation: white text on red background, 100% conserved; boxed red text on white background, 70% conserved. Abbreviations are the same as in Supplementary Figure 2. The TgMcrC and EcMcrC sequences are 13% identical and 27% similar across the whole protein and 20% identical and 40% similar across the finger domain alone.

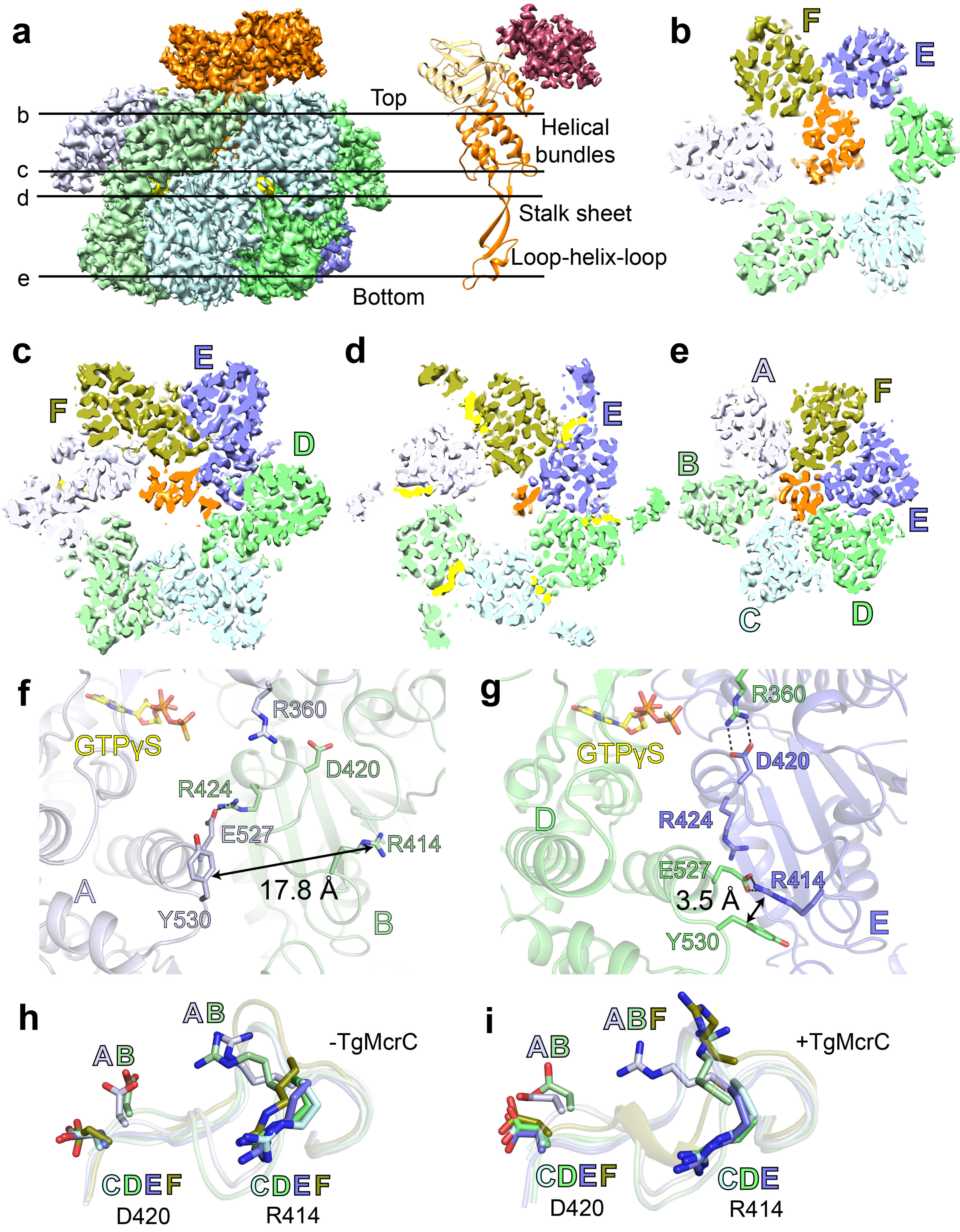


**Supplementary Figure 6: TgMcrC binding conforms to the intrinsic asymmetry of the TgMcrB hexamer.** (**a**) Side views of the TgMcrBC half-complex in surface representation (left panel) and TgMcrC in ribbon representation (right panel). (**b**-**e**) Slice sections through the density

at the levels indicated by the solid lines in (**a**). The TgMcrB subunits forming the main interactions with TgMcrC at the different levels are labeled. (**f-g**) Close-up views of interacting residues at the tight A/B interface (**f**) and the loose D/E interface (**g**) of the TgMcrB hexamer, shown from the same angles as in Figure 1d and e. (**h-i**) Superpositions of the 414-420 loop of the six subunits in TgMcrB by itself (**h**) and in TgMcrB in complex with TgMcrC (**i**).

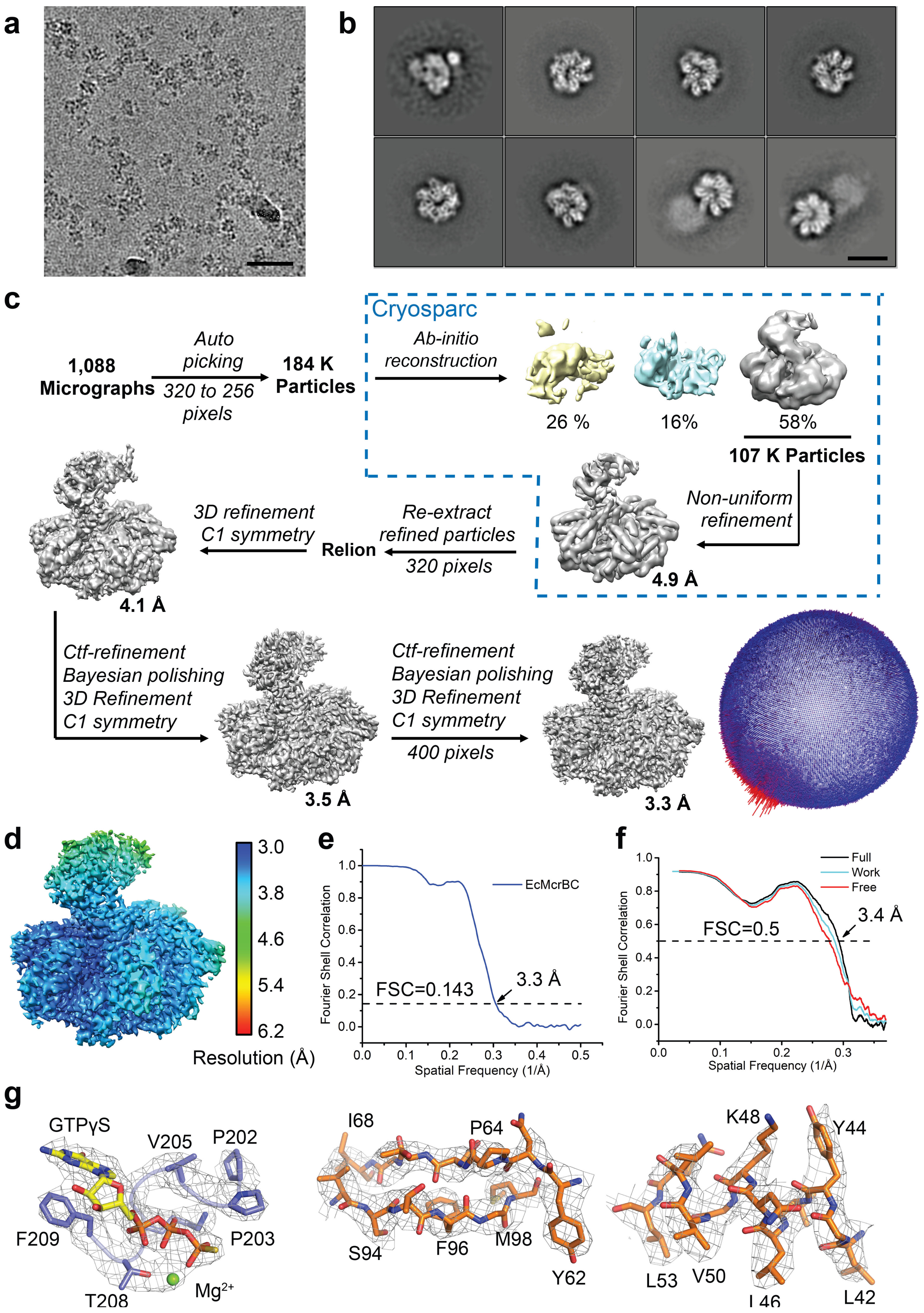
a
b
c
Cryosparc
1,088
Micrographs
Auto
picking
320 to 256
pixels
184 K
Particles
Ab-initio
reconstruction
26 %
16%
58%
107 K Particles
Non-uniform
refinement
4.9 Å
Re-extract
refined particles
320 pixels
Relion
3D refinement
C1 symmetry
4.1 Å
Ctf-refinement
Bayesian polishing
3D Refinement
C1 symmetry
3.5 Å
Ctf-refinement
Bayesian polishing
3D Refinement
C1 symmetry
400 pixels
3.3 Å
d
3.0
3.8
4.6
5.4
6.2
Resolution (Å)
e
EcMcrBC
FSC=0.143
3.3 Å
Fourier Shell Correlation
Spatial Frequency (1/Å)
f
Full
Work
Free
FSC=0.5
3.4 Å
Fourier Shell Correlation
Spatial Frequency (1/Å)
g
GTPγS
V205
P202
F209
P203
T208
Mg2+
I68
P64
S94
F96
M98
Y62
K48
Y44
L53
V50
L46
L42

**Supplementary Figure 7: Cryo-EM analysis of the EcMcrBC complex**. (**a**) Cryo-EM image area of vitrified EcMcrBC complexes. Scale bar is 50 nm. (**b**) Selected 2D-class averages of the EcMcrBC complex. Scale bar is 10 nm. (**c**) Cryo-EM data-processing workflow for the EcMcrBC complex and angular distribution plot of all EcMcrBC particles that contributed to the final map. The map and the angular distribution plot are shown in the same orientation. (**d**) Local resolution map for the EcMcrBC half-complex. (**e**) Gold-standard FSC curve for the EcMcrBC half-complex after correction for masking effects. The resolution was estimated based on the FSC = 0.143 criterion. (**f**) Cross-validation FSC curves for the EcMcrBC half-complex: cyan curve, refined model *versus* half map 1 used for refinement (Work); red curve, refined model *versus* half map 2 not used for refinement (Free); black curve, refined model *versus* the combined final map (Full). The similarity of the 'work' and 'free' curves suggests no substantial over-fitting. The correlation is above 0.5 up to a resolution of 3.4 Å. (**g**) Cryo-EM densities for selected regions of EcMcrB (left panel) and EcMcrC (middle and right panels) in the map of the EcMcrBC complex.

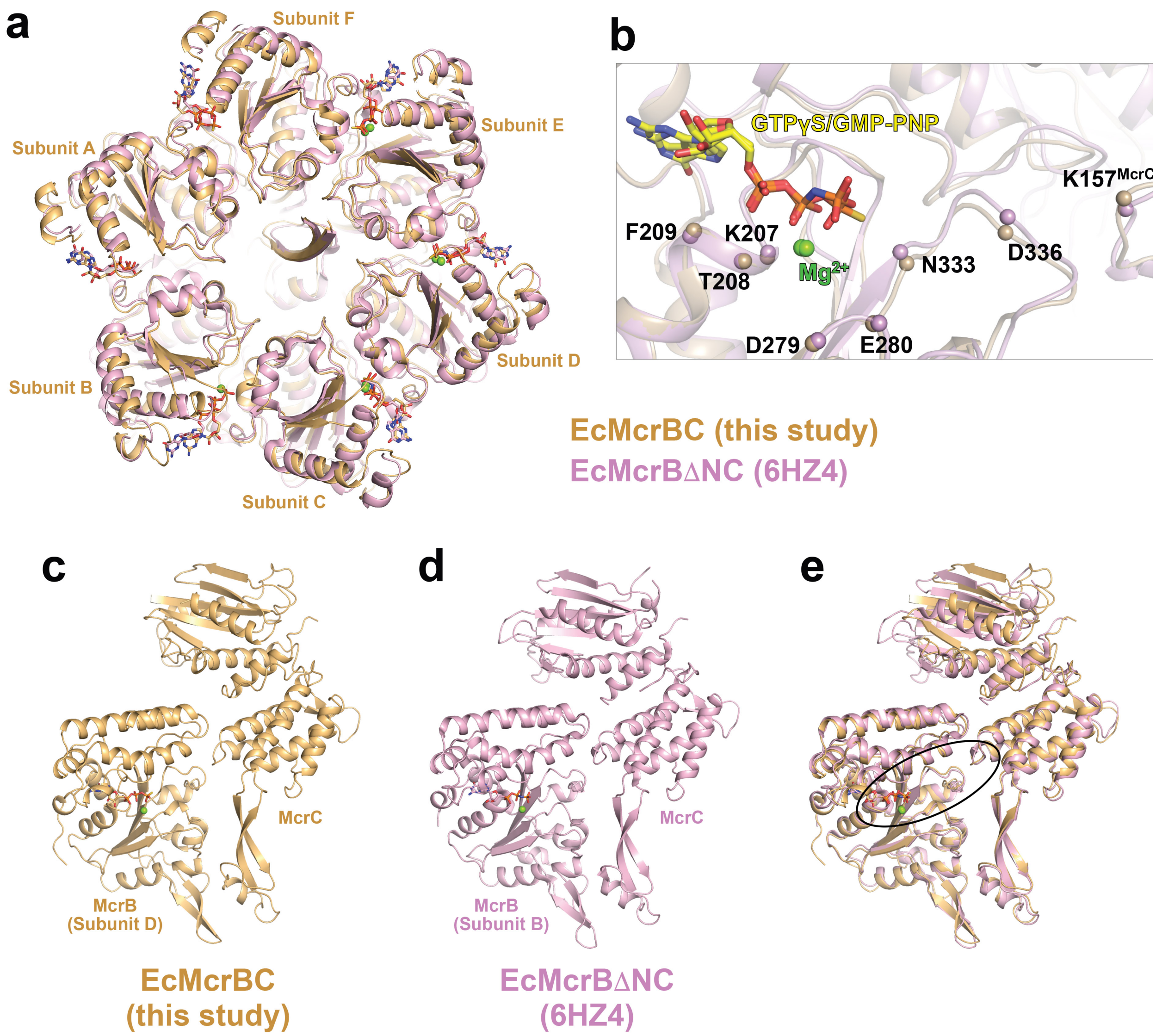


**Supplementary Figure 8: Structural comparison between the full-length EcMcrBC and the EcMcrBΔNC complexes.** (**a**) Superposition of the 'half' complexes of the *E. coli* McrBC structures determined in this study (light brown) and reported previously[1] (PDB: 6HZ4, light pink). A slice section at the level of the bound nucleotides is shown, viewed from the 'top' side of the McrB hexamers. (**b**) Close-up view of the GTP-binding site and the NxxD loop at the tight D/E interface in EcMcrBC, superimposed with the corresponding 'BC interface' in EcMcrBΔNC[1]. Each complex is colored as in (**a**). C$\alpha$ positions of the catalytically important residues are labeled and highlighted by spheres. Nucleotides and $Mg^{2+}$ ions are shown in ball-and-stick representation. (**c** and **d**) Side views of the McrC subunit and one of the McrB subunits in the half-complexes of EcMcrBC (**c**) and EcMcrBΔNC (**d**). (**e**) Superposition of the subunits depicted in (**c**) and (**d**). Oval line indicates the region shown in (**b**).

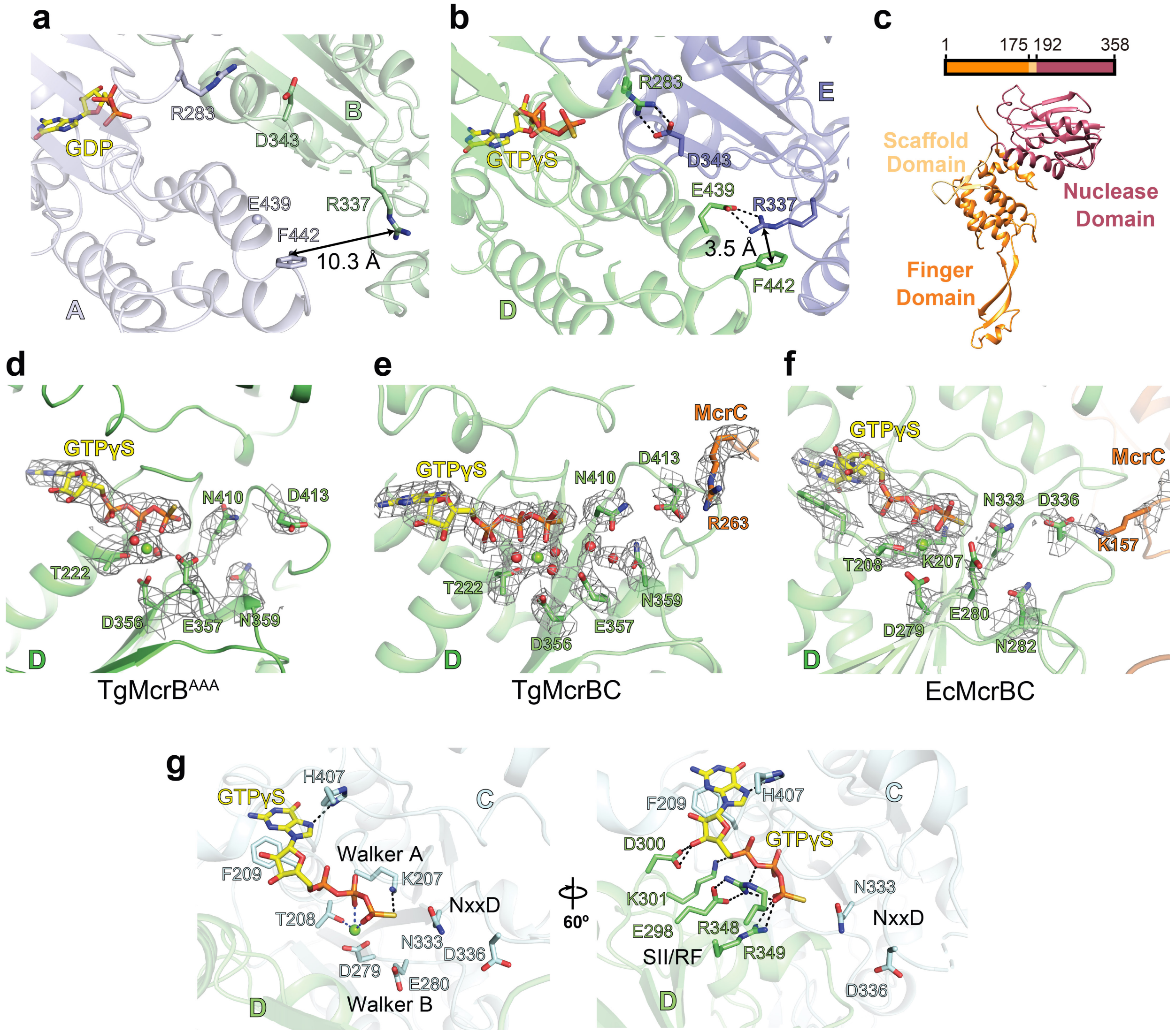


**Supplementary Figure 9: Cryo-EM structure of the full-length EcMcrBC complex.** (**a** and **b**) Close-up views of interacting residues at the loose A/B interface (**a**) and the tight D/E interface (**b**) of the EcMcrB hexamer. Black dashed lines indicate hydrogen bonds. (**c**) Domain architecture of EcMcrC. (**d-f**) Cryo-EM densities of the nucleotides/residues responsible for the hydrolysis stimulation in TgMcrB$^{AAA}$ (**d**), TgMcrBC (**e**), and EcMcrBC (**f**) shown in surface representation (grey mesh) overlaid with ball-and-stick representation. (**g**) Close-up views of the GTP-binding site at the tight C/D interface, highlighting *in cis* (left panel) and *in trans* (right panel) interactions. The Walker A, Walker B, NxxD, and Sensor II/arginine finger (SII/RF) motifs are labeled.

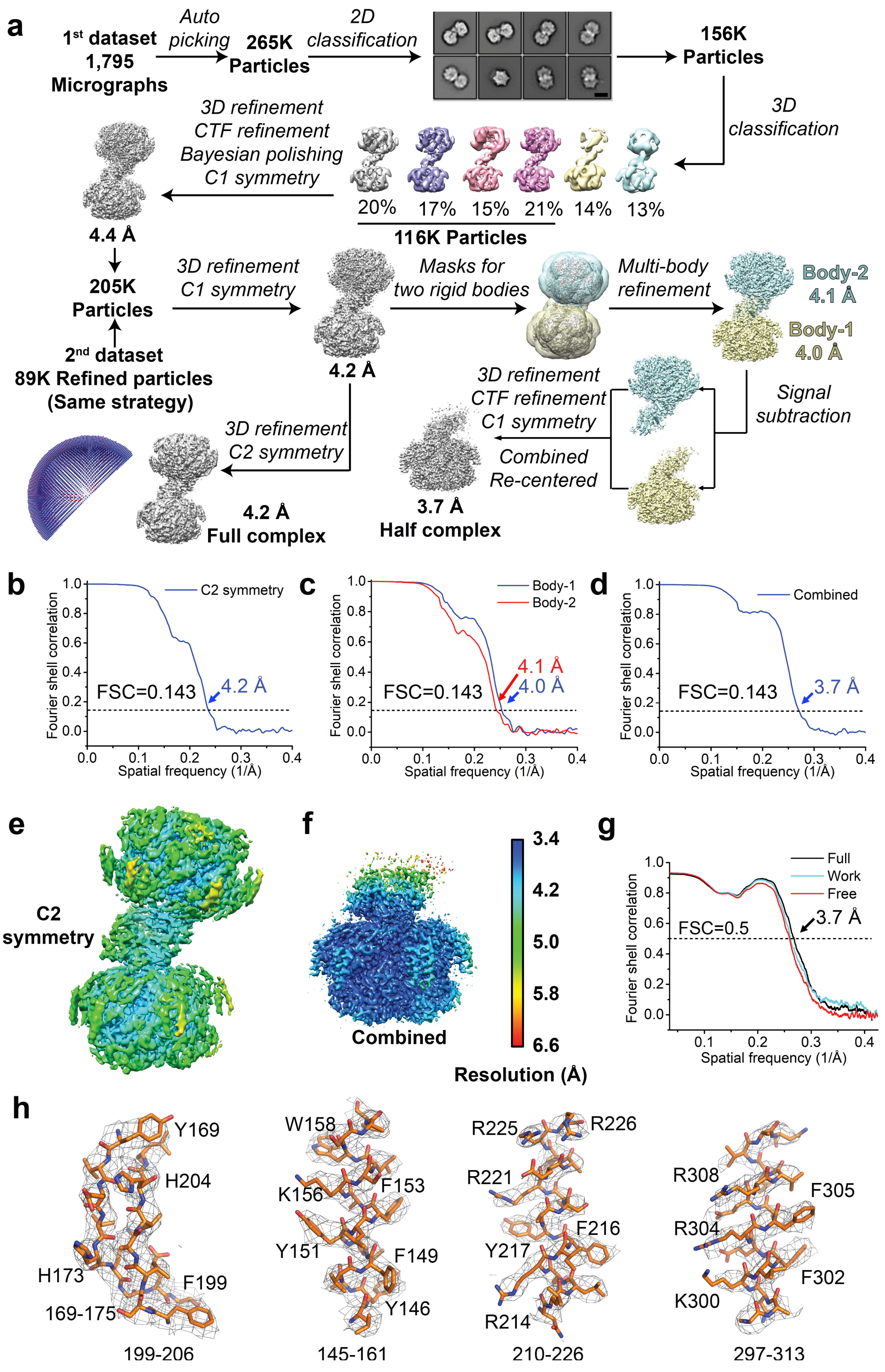

a
1st dataset
1,795
Micrographs
Auto
picking
265K
Particles
2D
classification
156K
Particles
3D
classification
3D refinement
CTF refinement
Bayesian polishing
C1 symmetry
20%
17%
15%
21%
14%
13%
116K Particles
4.4 Å
205K
Particles
2nd dataset
89K Refined particles
(Same strategy)
3D refinement
C1 symmetry
4.2 Å
Masks for
two rigid bodies
Multi-body
refinement
Body-2
4.1 Å
Body-1
4.0 Å
Signal
subtraction
3D refinement
CTF refinement
C1 symmetry
Combined
Re-centered
3.7 Å
Half complex
3D refinement
C2 symmetry
4.2 Å
Full complex
b
C2 symmetry
FSC=0.143
4.2 Å
Fourier shell correlation
Spatial frequency (1/Å)
c
Body-1
Body-2
FSC=0.143
4.1 Å
4.0 Å
Fourier shell correlation
Spatial frequency (1/Å)
d
Combined
FSC=0.143
3.7 Å
Fourier shell correlation
Spatial frequency (1/Å)
e
C2
symmetry
f
Combined
3.4
4.2
5.0
5.8
6.6
Resolution (Å)
g
Full
Work
Free
FSC=0.5
3.7 Å
Fourier shell correlation
Spatial frequency (1/Å)
h
Y169
H204
H173
F199
169-175
199-206
W158
K156
F153
Y151
F149
Y146
145-161
R225
R226
R221
F216
Y217
R214
210-226
R308
F305
R304
F302
K300
297-313

**Supplementary Figure 10: Cryo-EM analysis of the TgMcrB$^{AAA}$C complex.** (**a**) Cryo-EM data-processing workflow for the TgMcrB$^{AAA}$C complex and angular distribution plot of all TgMcrB$^{AAA}$C particles that contributed to the final map. The map and the angular distribution plot are shown in the same orientation. Scale bar is 10 nm. (**b–d**) Gold-standard FSC curves for the maps of the TgMcrB$^{AAA}$C complex obtained with different image-processing strategies after correction for masking effects: (**b**) the map obtained when C2 symmetry was imposed; (**c**) the maps of the two bodies obtained when multi-body refinement was used; and (**d**) the map obtained when the two half-complexes were combined. The resolution was estimated based on the FSC = 0.143 criterion. (**e** and **f**) Local resolution for the cryo-EM map with C2 symmetry imposed (**e**) and the map for the combined half-complexes (**f**). (**g**) Cross-validation FSC curves for the map of the combined TgMcrB$^{AAA}$C half-complexes: cyan curve, refined model *versus* half map 1 used for refinement (Work); red curve, refined model *versus* half map 2 not used for refinement (Free); black curve, refined model *versus* the combined final map (Full). The similarity of the 'work' and 'free' curves suggests no substantial over-fitting. The correlation is above 0.5 up to a resolution of 3.7 Å. (**h**) Cryo-EM densities for selected regions in the map of the combined TgMcrB$^{AAA}$C half-complexes.

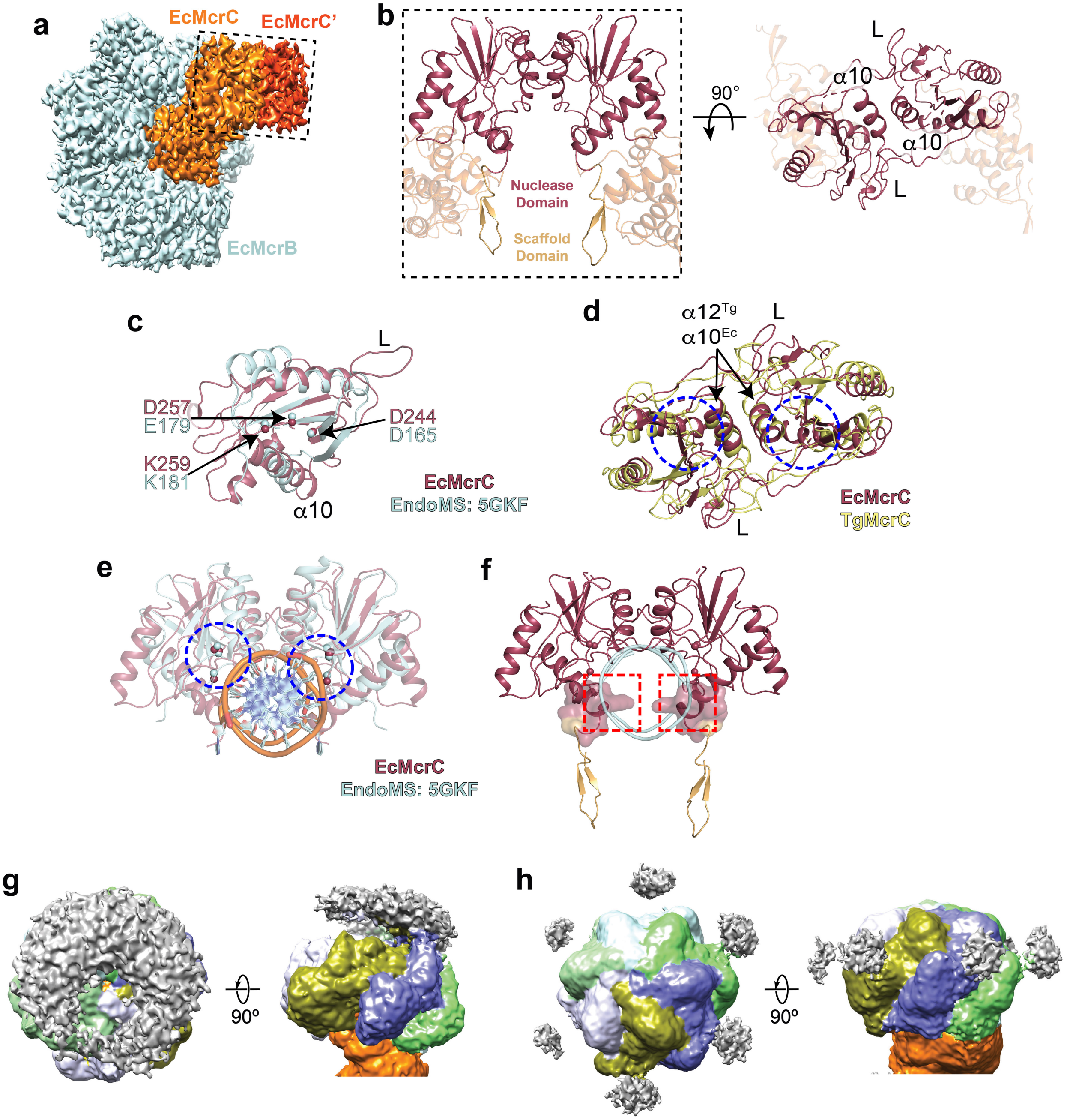


**Supplementary Figure 11**: **Structural comparison of the nuclease domains.** (**a**) Cryo-EM density map showing the interface between the two EcMcrC subunits (orange and red) and one EcMcrB hexamer (cyan). (**b**) Ribbon representation of the dimer interface of EcMcrC. (**c**) Structural comparison of an EcMcrC monomer with an EndoMS monomer (PDB: 5GKF), showing that the active-site residues (labeled spheres) overlap. (**d**) Superposition of the nuclease domains in the TgMcrC dimer (yellow) and EcMcrC dimer (red). The active sites for DNA cleavage are highlighted by blue circles. (**e**) Structural comparison of the EcMcrC nuclease-domain dimer in

the EcMcrBC complex (dark red) with the EndoMS dimer in a DNA-bound state (PDB: 5GKF) (cyan). The active sites for DNA cleavage are highlighted by blue circles. (**f**) Illustration of the cleavage-incompetent conformation of EcMcrC. For clarity, the EndoMS structure is not shown. The backbone of the DNA substrate bound to EndoMS is colored cyan. The red squares indicate regions of potential steric clashes. (**g** and **h**) Low-pass filtered maps of the EcMcrBC complex (**g**) and the TgMcrBC complex (**h**). Unassigned densities are colored in grey and likely represent the six N-terminal DNA-binding domains of the McrB hexamer.

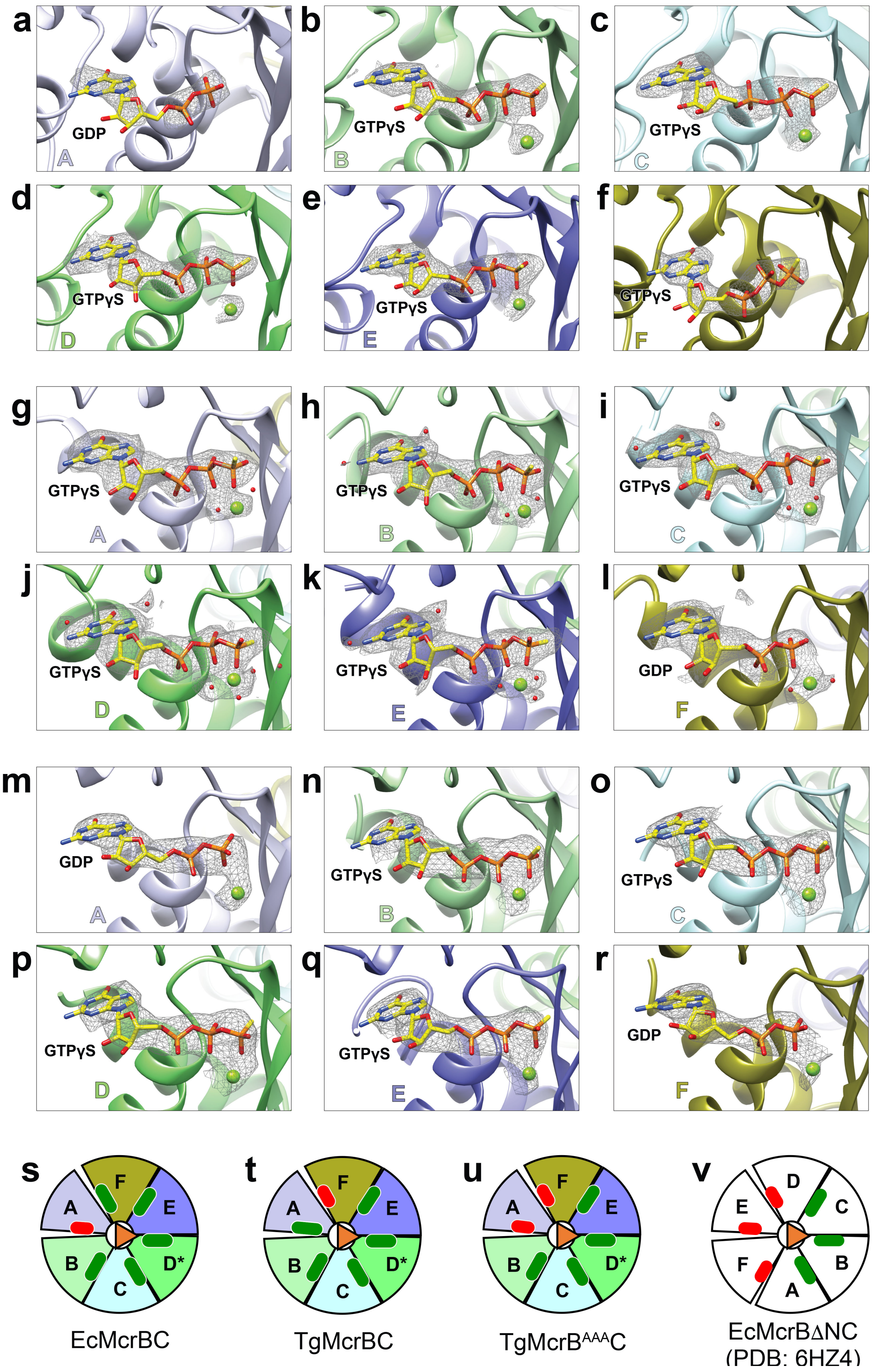
a
GDP
A
b
GTPγS
B
c
GTPγS
C
d
GTPγS
D
e
GTPγS
E
f
GTPγS
F
g
GTPγS
A
h
GTPγS
B
i
GTPγS
C
j
GTPγS
D
k
GTPγS
E
l
GDP
F
m
GDP
A
n
GTPγS
B
o
GTPγS
C
p
GTPγS
D
q
GTPγS
E
r
GDP
F
s
F
A
E
B
D*
C
EcMcrBC
t
F
A
E
B
D*
C
TgMcrBC
u
F
A
E
B
D*
C
TgMcrBAAAC
v
D
E
C
F
B
A
EcMcrBΔNC
(PDB: 6HZ4)

**Supplementary Figure 12: Assignment of the bound nucleotides in the GTP-binding sites of the McrB hexamers.** (**a**-**r**) Cryo-EM densities of the bound nucleotides, magnesium ions and water molecules at the hexameric McrB subunit interfaces in the EcMcrBC (**a**-**f**), TgMcrBC (**g**-**l**), and TgMcrB$^{AAA}$C (**m**-**r**) complexes, shown in surface representation (grey mesh) overlaid with ball-and-stick representation of the models. The subunits are colored as in Figure 5 (for EcMcrB) and Figure 3 (for TgMcrB). (**s**-**v**) Schematic diagrams of the subunit organization and the bound nucleotides in the structures of the EcMcrBC (**s**), TgMcrBC (**t**) and TgMcrB$^{AAA}$C (**u**) complexes in this study and the previously published structure of the EcMcrBΔNC complex[1] (**v**). Ovals at the interfaces of the hexamers represent the bound GTP analogs (green) and GDP (red). Asterisks represent the McrC-stimulating subunits.

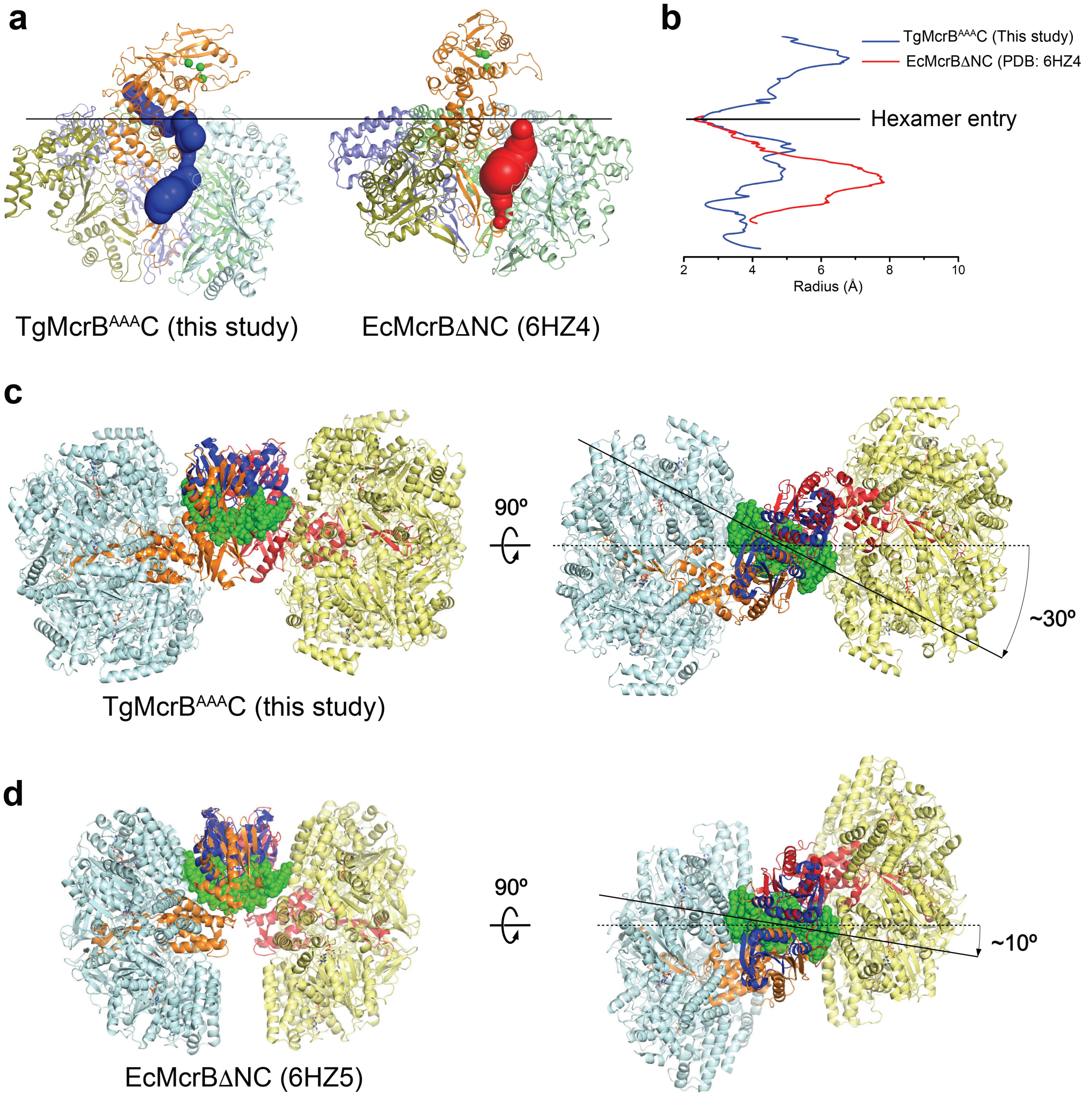


**Supplementary Figure 13: Hexamer pore accessibility and putative orientation of DNA substrates in McrBC complexes.** (**a**) Surface representation of the accessible pathway through the central pores of the TgMcrB$^{AAA}$C and EcMcrBΔNC hexamers, calculated by using MOLEonline[2]. The A subunits are omitted from each structure for clarity. (**b**) Radius plot of the accessible pathway shown in (**a**). The black line indicates the possible entry point into each hexamer in (**a**). (**c** and **d**) Two different side views of the tetradecameric TgMcrB$^{AAA}$C (**c**, in this study) and the EcMcrBΔNC (**d,** PDB: 6HZ5) complexes, colored as in Figure 6a. Both complexes are superimposed with the dimeric nuclease domains (blue ribbon) and the bound DNA (green

spheres) from the EndoMS crystal structure[3] (PDB: 5GKF). Dashed lines indicate the axes perpendicular to the ring planes of the two opposing McrB hexamers with the angles of the superimposed DNA strands relative to these axes labeled.

## Supplementary Table 1. Sequences of the codon-optimized genes used in this study

| Name | Sequence |
|---|---|
| TgMcrB | ATGGAAAACCAGCTGTTTATTATCGGTATTGGCACCGGCACCGATGAATATGAAAATTTTGAAGAAACCATCCTGAAAGGCGTGAAACGTAATGAACTGGAAGGTCAGATT<br>GGTCCGGATATTCTGGATAATTGTTGTTCCGATGTGTGCTATTTTTGGGGTCGTAGCAAAGAAACGATCTATGAGAAAAAAATCGATAAAGGCGACATGGTGCTGTTCTATG<br>TTGGTAAACGTATTAGCCGCAATAAAGTGGACCTGAATCAAGAAACCGCAGTGTATCTGGGTATTATTTGTGAAACCGTGGAAATCAGCGAAAACGATGTTAGCTTTCTGAA<br>TGATTTTTGGCGCAAAGGTGAAAACTTTCGCTTTCTGATGTTCTTTAAAAAAAAACCGGAAAAACTGCACCACAGCATCAACGAAATTAACAGCAAACTGGGTTACAACCCG<br>GATTATTTTCCGATTGCAGGTTATGTTAAACCGGAACGTATGAGCGGTGTTTACGATATTCTGAAAAATATCCTGAAAAAACGTGGCATTCTGAAAGAAAGCGATAGCATGA<br>ATGAAAGCGCAGGCCATAACATCAAAGAAGATTATTTCCGCGTTGACATGCTGCTGAACAAAAAAGGTCAGGTTATTCTGTATGGTCCGCCTGGCACCGGTAAAACCTGGA<br>TTGCACGTAAATATGTTGTGGAAGAAACGAACGAAAAAACCCCTGGTAATAAATGGGAGTTTATCACGTTTCATCAGAGCTACAGCTATGAAGAATTTATCGAAGGTTTTCG<br>TCCGCGTACGGATAATGAAGAAAAAATTCGTTATGTGGTCGAGGATGGCATTTTCAAAAAAATCGCACTGCGTGCACTGGTGAAAGGTCTGTTTGAGCTGGAAGATGCAAC<br>CATTGGCAAAGATAAAATCCACCGTCTGTATATCCTGCTGACGAAAAAAGAACCGCTGAGCCCGACCGAATATGAAGAGTATCTGCGTCTGAAACGTTATCTGTGGGAACT<br>GGTTGGTGGTCTGCCGAAAGATAAACTGAAAAATCTGACCCCGAAATTCTACCTGATTATCGATGAAATTAACCGTGGCAACATTAGCAAAATCTTTGGCGAACTGATTACC<br>CTGCTGGAAAAAGACAAACGTCTGGGTGGTGAAAATCAACTGATTGTTCGTCTGCCGTATAGCGGTGAACCGTTTGCAGTTCCGCCTAATCTGTACATTATTGGTACAATG<br>AATACCGCAGATCGTAGCATTGCACTGCTGGATGTTGCACTGCGTCGTCGTTTTGCATTTATTGAAGTTGAACCGCGTCCGGAATTTCTGGAAAAAGAGAATCTGAAAAAA<br>ATCCGCGAGAAAAAACTGAAAACCGAAGATCGTAAACGCCTGAACGAGAAACTGAATGAACTGTTTAGTAAACTGGGCAACGATAACTACTTTCTGAAAACGCTGCTGGAA<br>AAAATCAATGTGCGTATTACCGTTGTGAAAGATCGTGATCATCGTATTGGCCATAGCTATTTTCTGAACGTTGAAACCGTTGAAGATCTGCATCATGTGTGGTATTATGAAG<br>TTCTGCCGCTGCTGATGGAATATTTCTATAATGATTGGGAAACCATCAAATGGGTGCTGAATGAAAAAGGCAAAGAACATGGCAACGTGTTTTTTGAGAAACTGCGCCTGA<br>CCGGTCCGAATGGTGAAGAAGCATATCAGCTGAAAGTTCTGGAAGGTGATGCCTTTATTGGTGCACTGAAACGCATTATCAGCAAAAATACCCCGAGCCAAGAAGGTGGC<br>GCAACCACCAACGAAGAAAATAGTCCGGAAAACACCCAGAGCCAGACCGAAGGTGAT |
| TgMcrC | ATGCCTCGTCTGACCACCATTACCCTGTATGAACATGATGAAAAACGCTATCGTGATATCGCAGGCGATAAAAAAGCAATTCAGGATGCACTGATCAAACTGAACAAACAG<br>TTCAAAAAAGATTTCAAAAAACTGGACCGCAGCGAGGATAATAGCGATACCGAAGATACCATTGATGAAAGCAAAGGTGTTGTTGAAGTGTATGCCAACAAAATCAAAGCC<br>CGTCATTATGTTGGTTTTGCAGCCGTTGATAATGTGTTTCTGCAGATTCTGCCGAAAGTGTTTAAACCGAAAAAAGAGCAGACCCAAGAAACGCAAGAAGATACCTGGGAA<br>CCGATTCTGGCATTTATTCGTATGCTGGATATGGCCTATGGCCTGAAAATCAAAGATCACGATCTGGCATATCTGCAGGGTCGTAATCTGCGTCCGAATCTGTATGAAGTG<br>TTTATTTACCTGTTTGCCAAAAGCCTGTGGTCTGAAGTTCAGCGTGGTTATCATCGTGAATATGTTGAAGTACACCGCGAAGAAAAATTTCTGCGTGGTAAACTGCTGATGA<br>GCCGTCAGATTCGTAAACTGCCGCATCAGCTGAATACCTTTAGCGTTGAAGTGCATGAACTGATCGAAGATAATCTGCTGAACCGTATCTTTTATGCAAGCGTTCGTGAAG<br>CACTGCGTCGTACCACCTGGGGTCTGAATCGCAAACTGCTGGGTGAACTGATGCTGGCCTTTGATGGTATTACCCCGATTCATCTGCGCACCGAACATTTTGAACGTGTT<br>CATTTTACCCGTCTGAATGAACGTTTTCGTCGTCCGTTTGAACTGGCAAAACTGCTGTTTATGCCTGCAAGCGGTAAAGGTCGTAGCCGTGAAGTTAGCGGTTTTTTTGTG<br>GATATGAACAAACTGTTTGAGCGCTTTATTGAACGCGTGCTGGTTCGCAATCTGCCTCCGGAATATAAACTGTTTTATCAAGAAAGCTATCCGTTTCTGAAAAACCAGAATG<br>GTAGCAGCCAGAAACCGGATTATGTTGTTCGTAAAGGTAATACACCGGTTGTTGTTCTGGATGCCAAATATCGTGAACTGAAAGAACGTATTCCGAGCAGCGATATGCTGC<br>GTCAGCTGTATGTTTATAGCCGTATTTGGGGTTATAAAACCAGCCATGAAAACGATAGCAAACCGCCTGCAGTTATTGTTATTCCGAGTAGCAGCACCTATAATCAGGGTCT<br>GCCGGATAAACCGCTGGAATTTGAATTTTTTGATGAGCGCAAACTGTTCATCGTGGCCTATAATATGGATTACGTTAAAACCGGTGCCATCTTCAAAGCCGATAAAAACTTT<br>CGTCGTTCCCTGAACAACATTATCGGTAAACTGAATACC |
| EcMcrB | ATGGAAAGCATTCAGCCGTGGATCGAGAAATTTATCAAACAGGCACAGCAGCAGCGTAGCCAGAGCACCAAAGATTATCCGACCAGCTATCGTAATCTGCGTGTTAAACT<br>GAGCTTTGGCTATGGTAATTTTACCAGCATTCCGTGGTTTGCATTTCTGGGTGAAGGTCAAGAAGCAAGCAACGGTATTTATCCGGTTATCCTGTATTACAAAGATTTCGAT<br>GAACTGGTTCTGGCCTATGGTATTAGCGATACCAATGAACCGCATGCACAGTGGCAGTTTAGCAGCGATATTCCGAAAACCATTGCCGAATATTTTCAGGCAACCAGCGGT<br>GTTTATCCGAAAAAATACGGTCAGAGCTATTATGCCTGTAGCCAGAAAGTTAGCCAGGGTATTGATTATACCCGTTTTGCAAGCATGCTGGACAACATCATCAACGATTACA<br>AACTGATTTTTAACAGCGGCAAAAGCGTTATTCCGCCTATGAGCAAAACCGAAAGCTATTGTCTGGAAGATGCACTGAATGACCTGTTTATTCCGGAAACAACCATTGAAAC<br>CATTCTGAAACGTCTGACCATCAAAAAAAACATCATTCTGCAGGGTCCGCCTGGTGTTGGTAAAACCTTTGTTGCACGTCGTCTGGCATATCTGCTGACCGGTGAAAAAGC<br>ACCGCAGCGTGTTAATATGGTTCAGTTTCATCAGAGCTACAGCTATGAAGATTTTATCCAGGGTTATCGTCCGAATGGTGTGGGTTTTCGTCGTAAAGATGGCATCTTCTAT<br>AACTTTTGTCAGCAGGCAAAAGAGCAGCCGGAAAAAAAATATATCTTCATCATCGACGAAATCAACCGTGCCAATCTGAGCAAAGTTTTTGGTGAAGTTATGATGCTGATGG<br>AACATGATAAACGCGGTGAAAATTGGAGCGTTCCGCTGACCTATAGCGAAAACGATGAAGAACGTTTTTATGTGCCGGAAAACGTGTATATTATCGGCCTGATGAATACCG<br>CAGATCGTAGCCTGGCAGTTGTTGATTATGCACTGCGTCGTCGTTTTAGCTTCATTGATATTGAACCGGGTTTTGATACACCGCAGTTTCGTAATTTTCTGCTGAACAAAAA<br>AGCCGAACCGAGCTTTGTTGAAAGCCTGTGTCAGAAAATGAACGAGCTGAATCAAGAAATCAGCAAAGAAGCCACCATTCTGGGTAAAGGCTTTCGTATTGGTCATAGCTA<br>TTTTTGTTGTGGCCTGGAAGATGGCACCAGTCCGGATACCCAGTGGCTGAATGAAATTGTTATGACCGATATTGCACCGCTGCTGGAAGAATATTTCTTTGATGATCCGTA<br>TAAACAGCAGAAATGGACCAATAAACTGCTGGGTGATAGC |
| EcMcrC | ATGGAACAGCCGGTTATTCCGGTTCGCAATATCTATTACATGCTGACCTATGCATGGGGCTATCTGCAAGAAATTAAACAGGCAAATCTGGAAGCAATTCCGGGTAATAAT<br>CTGCTGGATATTCTGGGTTATGTGCTGAATAAAGGTGTTCTGCAGCTGAGCCGTCGTGGTCTGGAACTGGATTATAACCCGAATACCGAAATTATCCCTGGTATTAAAGGT<br>CGCATCGAATTTGCAAAAACCATCCGTGGTTTTCATCTGAATCATGGTAAAACCGTTAGCACCTTTGATATGCTGAATGAAGATACCCTGGCCAACCGTATTATCAAAAGCA<br>CCCTGGCAATTCTGATCAAACACGAAAAACTGAATAGCACCATTCGTGATGAAGCACGTAGCCTGTATCGTAAACTGCCAGGTATTAGCACCCTGCATCTGACACCGCAGC<br>ATTTTTCATATCTGAATGGTGGCAAAAACACCCGCTATTACAAATTCGTTATTAGCGTGTGCAAATTCATCGTGAATAATAGCATTCCGGGTCAGAACAAAGGTCACTATCG<br>CTTTTATGATTTTGAACGCAACGAGAAAGAAATGAGCCTGCTGTATCAGAAATTCCTGTATGAATTTTGTCGTCGTGAACTGACCAGCGCAAATACCACCCGTAGCTATCTG<br>AAATGGGATGCAAGCAGCATTAGCGATCAGAGCCTGAATCTGCTGCCTCGTATGGAAACCGATATTACCATTCGTAGCAGCGAAAAAATCCTGATTGTTGACGCCAAATAC<br>TATAAAAGCATTTTCAGCCGTCGTATGGGCACCGAAAAATTTCATAGCCAGAATCTGTACCAGCTGATGAATTATCTGTGGTCACTGAAACCGGAAAACGGTGAAAATATTG<br>GTGGTCTGCTGATTTATCCGCATGTTGATACCGCAGTTAAACACCGCTATAAAATCAACGGTTTTGATATTGGTCTGTGCACCGTTAATCTGGGTCAAGAATGGCCGTGTAT<br>TCATCAAGAACTGCTGGACATTTTTGATGAGTACCTGAAA |

**Supplementary Table 2. Primers used in this study**

| Name | Sequence (5' to 3') |
|---|---|
| TgMcrB R360A | TTCTACCTGATTATCGATGAAATTAACgcaGGCAACATTA |
| TgMcrB R360A reverse | TAATGTTGCCTGCGTTAATTTCATCGATAATCAGGTAGAA |
| TgMcrB R414A | TACAATGAATACCGCAGATgcaAGCATTGCACTGCTGGAT |
| TgMcrB R414A reverse | ATCCAGCAGTGCAATGCTTGCATCTGCGGTATTCATTGTA |
| TgMcrB D420A | CGTAGCATTGCACTGCTGgcaGTTGCACTGCGTCGTCGTT |
| TgMcrB D420A reverse | AACGACGACGCAGTGCAACTGCCAGCAGTGCAATGCTACG |
| TgMcrB R424A | CTGCTGGATGTTGCACTGgcaCGTCGTTTTGCATTTATTG |
| TgMcrB R424A reverse | CAATAAATGCAAAACGACGTGCCAGTGCAACATCCAGCAG |
| TgMcrB Y530A | CTGCTGATGGAATATTTCgcaAATGATTGGGAAACCATCA |
| TgMcrB Y530A reverse | TGATGGTTTCCCAATCATTTGCGAAATATTCCATCAGCAG |
| TgMcrB K221A | GGTCCGCCTGGCACCGGTgcaACCTGGATTGCACGTAAAT |
| TgMcrB K221A reverse | ATTTACGTGCAATCCAGGTTGCACCGGTGCCAGGCGGACC |
| TgMcrB T222A | CCGCCTGGCACCGGTAAAgcaTGGATTGCACGTAAATATG |
| TgMcrB T222A reverse | CATATTTACGTGCAATCCATGCTTTACCGGTGCCAGGCGG |
| TgMcrB W223A | CCTGGCACCGGTAAAACCgcaATTGCACGTAAATATGTTG |
| TgMcrB W223A reverse | CAACATATTTACGTGCAATTGCGGTTTTACCGGTGCCAGG |
| TgMcrB D356A | AAATTCTACCTGATTATCgcaGAAATTAACCGTGGCAACA |
| TgMcrB D356A reverse | TGTTGCCACGGTTAATTTCTGCGATAATCAGGTAGAATTT |
| TgMcrB E357A | TTCTACCTGATTATCGATgcaATTAACCGTGGCAACATTA |
| TgMcrB E357A reverse | TAATGTTGCCACGGTTAATTGCATCGATAATCAGGTAGAA |
| TgMcrB E375A | GAACTGATTACCCTGCTGgcaAAAGACAAACGTCTGGGTG |
| TgMcrB E375A reverse | CACCCAGACGTTTGTCTTTTGCCAGCAGGGTAATCAGTTC |
| TgMcrB D377A | ATTACCCTGCTGGAAAAAgcaAAACGTCTGGGTGGTGAAA |
| TgMcrB D377A reverse | TTTCACCACCCAGACGTTTTGCTTTTTCCAGCAGGGTAAT |
| TgMcrB K378A | ACCCTGCTGGAAAAAGACgcaCGTCTGGGTGGTGAAAATC |
| TgMcrB K378A reverse | GATTTTCACCACCCAGACGTGCGTCTTTTTCCAGCAGGGT |
| TgMcrB N410A | TACATTATTGGTACAATGgcaACCGCAGATCGTAGCATTG |
| TgMcrB N410A reverse | CAATGCTACGATCTGCGGTTGCCATTGTACCAATAATGTA |
| TgMcrB D413A | GGTACAATGAATACCGCAgcaCGTAGCATTGCACTGCTGG |
| TgMcrB D413A reverse | CCAGCAGTGCAATGCTACGTGCTGCGGTATTCATTGTACC |

| | |
|---|---|
| TgMcrB R425A | CTGGATGTTGCACTGCGTgcaCGTTTTGCATTTATTGAAG |
| TgMcrB R425A reverse | CTTCAATAAATGCAAAACGTGCACGCAGTGCAACATCCAG |
| TgMcrB R426A | GATGTTGCACTGCGTCGTgcaTTTGCATTTATTGAAGTTG |
| TgMcrB R426A reverse | CAACTTCAATAAATGCAAATGCACGACGCAGTGCAACATC |
| TgMcrB H501A | CGTGATCATCGTATTGGCgcaAGCTATTTTCTGAACGTTG |
| TgMcrB H501A reverse | CAACGTTCAGAAAATAGCTTGCGCCAATACGATGATCACG |
| TgMcrB N359A | TTCTACCTGATTATCGATGAAATTgcaCGTGGCAACATTA |
| TgMcrB N359A reverse | TAATGTTGCCACGTGCAATTTCATCGATAATCAGGTAGAA |
| TgMcrC R263A | GAACGTGTTCATTTTACCGCACTGAATGAACGTTTTCGTC |
| TgMcrC R263A reverse | GACGAAAACGTTCATTCAGTGCGGTAAAATGAACACGTTC |
| TgMcrC R263K | GAACGTGTTCATTTTACCAAACTGAATGAACGTTTTCGTC |
| TgMcrC R263K reverse | GACGAAAACGTTCATTCAGTTTGGTAAAATGAACACGTTC |

**Supplementary Table 3. X-ray data collection and refinement statistics.**

| | TgMcrB$^{AAA}$ Native | TgMcrB$^{AAA}$ Se derivative |
|---|---|---|
| **PDB ID** | 6UT3 | |
| **Data collection** | | |
| X-ray Source | NECAT 24ID-E | NECAT 24ID-C |
| Wavelength (Å) | 0.9791 | |
| Space Group | $P2_1$ | |
| Cell dimensions | | |
| *a*, *b*, *c* (Å) | 100.02, 108.55, 118.43 | 100.24, 108.87, 118.67 |
| α, β, γ (°) | 90, 106.94, 90 | 90, 107.41, 90 |
| Resolution (Å) | 114.650-2.95 (3.04-2.95) | 113.23-3.14 (3.25-3.14) |
| $R_{merge}$ (%) | 7.3 (141.5) | 10.6 (138.6) |
| $R_{meas}$ (%) | 7.9 (167.4) | 11.1 (149.5) |
| $CC_{1/2}$ (%) | 99.9 (55.6) | 99.9 (73.8) |
| $I / \sigma I$ | 14.2 (1.0) | 17.3 (1.9) |
| Completeness (%) | 99.6 (99.8) | 99.7 (99.9) |
| Redundancy | 6.8 (6.9) | 13.5 (14.0) |
| **Phasing** | | |
| Initial F.O.M. | | 0.499 |
| Number of sites | | 19 |
| **Refinement** | | |
| Resolution (Å) | 114.65-2.95 | |
| No. reflections | 53,315 (4,037) | |
| $R_{work}$ / $R_{free}$ (%) | 34.5/36.4 | |
| Number of atoms | | |
| Protein | 15915 | |
| Ligand/ion | 132 | |
| *B*-factors | | |
| Protein | 128.5 | |
| Ligand/ion | 110.4 | |
| Clash score | 13.4 | |
| R.m.s. deviations | | |
| Bond lengths (Å) | 0.007 | |
| Bond angles (°) | 1.21 | |
| Ramachandran plot | | |
| Favored (%) | 94.4 | |
| Allowed (%) | 5.6 | |
| Outliers (%) | 0 | |

*Values in parentheses are for highest-resolution shell. Each dataset was derived from a single crystal.

## Supplementary Table 4. Cryo-EM data collection and refinement statistics.

| | EcMcrBC | TgMcrB$^{AAA}$ | TgMcrBC | TgMcrB$^{AAA}$C (Full mask) | TgMcrB$^{AAA}$C (Body 1) | TgMcrB$^{AAA}$C (Body 2) | TgMcrB$^{AAA}$C (Combined) |
|---|---|---|---|---|---|---|---|
| **EMDB ID** | EMD-20867 | EMD-20865 | EMD-20866 | EMD-20868 | EMD-20869 | EMD-20870 | EMD-20871 |
| **PDB ID** | 6UT6 | 6UT4 | 6UT5 | 6UT7 | | | 6UT8 |
| **Data collection** | | | | | | | |
| Microscope | Titan Krios | | | | | | |
| Detector | K2 summit | | | | | | |
| Voltage (kV) | 300 | | | | | | |
| Pixel size (Å) | 0.50 | | | | | | |
| Total electron exposure (e$^-$/Å$^2$) | 80.0 | | | | | | |
| Defocus range (μm) | -1.0 to -3.0 | -1.5 to -3.0 | -1.5 to -2.5 | -2.0 to -3.5 | | | |
| Micrographs collected | 1,161 | 1,599 | 2,078 | 4,271 | | | |
| **Reconstruction** | | | | | | | |
| Final particle images | 106,684 | 139,306 | 226,846 | 204,593 | | | 409,186 |
| Pixel size (Å) | 1.0 | 1.25 | 1.0 | 1.25 | | | |
| Box size (pixels) | 400 | 256 | 400 | 256 | | | |
| Resolution (Å) (FSC = 0.143) | 3.28 | 3.14 | 2.44 | 4.26 | 3.95 | 4.10 | 3.68 |
| Map Sharpening B-factor (Å) | -45.2 | -51.1 | -20.6 | -160.4 | -138.2 | -154.8 | -126.9 |
| **Model composition** | | | | | | | |
| Non-hydrogen atoms | 16,938 | 19,126 | 22,374 | 45,062 | | | 22.531 |
| Protein residues | 2,078 | 2,315 | 2,697 | 5,522 | | | 2,761 |
| Water | 0 | 12 | 28 | 0 | | | 0 |
| Ligands | 5 (GTPγS) 1 (GDP) | 6 (GTPγS) | 5 (GTPγS) 1 (GDP) | 8 (GTPγS) 4 (GDP) | | | 4 (GTPγS) 2 (GDP) |
| Metals | 4 ($Mg^{2+}$) | 6 ($Mg^{2+}$) | 6 ($Mg^{2+}$) | 12 ($Mg^{2+}$) | | | 6 ($Mg^{2+}$) |
| **Refinement** | | | | | | | |
| Model-to-map CC (mask) | 0.78 | 0.84 | 0.82 | 0.68 | | | 0.80 |
| Model-to-map CC (volume) | 0.77 | 0.82 | 0.81 | 0.72 | | | 0.77 |
| R.m.s deviations | | | | | | | |
| Bond length (Å) | 0.005 | 0.004 | 0.005 | 0.004 | | | 0.004 |
| Bond angles (°) | 0.819 | 0.740 | 0.780 | 0.832 | | | 0.831 |
| **Validation** | | | | | | | |
| MolProbity score | 1.74 | 1.58 | 1.48 | 1.68 | | | 1.66 |
| Clash score | 7.39 | 5.89 | 5.90 | 5.38 | | | 5.08 |
| Ramachandran plot | | | | | | | |
| Outliers (%) | 0.0 | 0.0 | 0.0 | 0.0 | | | 0.0 |
| Allowed (%) | 4.8 | 3.8 | 2.9 | 5.8 | | | 5.8 |
| Favored (%) | 95.2 | 96.2 | 97.1 | 94.2 | | | 94.2 |
| Rotamer outliers (%) | 0.57 | 0.15 | 0.17 | 0.44 | | | 0.44 |
| C-beta deviations (%) | 0.05 | 0.05 | 0.00 | 0.00 | | | 0.00 |

**Supplementary References**


1. Nirwan, N. *et al.* Structure-based mechanism for activation of the AAA+ GTPase McrB by the endonuclease McrC. *Nat Commun* **10**, 3058 (2019).
2. Pravda, L. *et al.* MOLEonline: a web-based tool for analyzing channels, tunnels and pores. *Nucleic Acids Res* **46**, W368-W373 (2018).
3. Nakae, S. *et al.* Structure of the EndoMS-DNA Complex as Mismatch Restriction Endonuclease. *Structure* **24**, 1960-1971 (2016).
