# Supplementary material for: Structural asymmetry governs the assembly and GTPase activity of McrBC restriction complexes
Source: Nat Commun. 2020 Nov 20;11:5907. doi: 10.1038/s41467-020-19735-4 (PMC7680126; doi:10.1038/s41467-020-19735-4)
Supplement: Supplementary file 3 — Reporting Summary [file 41467_2020_19735_MOESM3_ESM.pdf]

nature research

Corresponding author(s): Joshua S. Chappie and Thomas Walz

Last updated by author(s): 2020/10/10

Reporting Summary

Nature Research wishes to improve the reproducibility of the work that we publish. This form provides structure for consistency and transparency in reporting. For further information on Nature Research policies, seeAuthors & Referees and theEditorial Policy Checklist .

Statistics

For all statistical analyses, confirm that the following items are present in the figure legend, table legend, main text, or Methods section.

n/a	Confirmed	
☐	☒	The exact sample size (*n*) for each experimental group/condition, given as a discrete number and unit of measurement
☐	☒	A statement on whether measurements were taken from distinct samples or whether the same sample was measured repeatedly
☒	☐	The statistical test(s) used AND whether they are one- or two-sided<br>*Only common tests should be described solely by name; describe more complex techniques in the Methods section.*
☒	☐	A description of all covariates tested
☒	☐	A description of any assumptions or corrections, such as tests of normality and adjustment for multiple comparisons
☐	☒	A full description of the statistical parameters including central tendency (e.g. means) or other basic estimates (e.g. regression coefficient) AND variation (e.g. standard deviation) or associated estimates of uncertainty (e.g. confidence intervals)
☒	☐	For null hypothesis testing, the test statistic (e.g. *F*, *t*, *r*) with confidence intervals, effect sizes, degrees of freedom and *P* value noted<br>*Give P values as exact values whenever suitable.*
☒	☐	For Bayesian analysis, information on the choice of priors and Markov chain Monte Carlo settings
☒	☐	For hierarchical and complex designs, identification of the appropriate level for tests and full reporting of outcomes
☒	☐	Estimates of effect sizes (e.g. Cohen's *d*, Pearson's *r*), indicating how they were calculated

Our web collection on statistics for biologists contains articles on many of the points above.

Software and code

Policy information about availability of computer code

Data collection	Cryo-EM data was automatically collected using Serial EM v3.6.<br>X-ray data collection was carried out remotely using the NE-CAT RAPD pipeline v2.0.1 for autoindexing and strategy incorporating LABELIT (in PHENIX v1.14), RADDOSE v2, BEST v3.4.4, Mosflm v.7.2.2, and Xtriage (in PHENIX v1.14).
Data analysis	RELION 3.0, MotionCor2 v2.3.2, Gctf v1.0.6, CtfFind v4.1.8, Gautomatch v0.53, CryoSPARC v2.4.0, and Pyem v0.4 were used for cryo-EM image processing.<br>XDS (BUILT=20180319) and AIMLESS v0.05.32 (as part of the NE-CAT RAPD pipeline v.2.0.1) were used to process collected X-ray diffraction data.<br>The Collaborative Computational Project Number 4 (CCP4) suite of crystallographic software v7.0 was used as noted below:<br>BLEND (in CCP4 v7.0), SHELX (in CCP4 v7.0), and CRANK2 (in CCP4 v7.0)were used for SAD phasing.<br>Phaser (in CCP4 v7.0) was used for molecular replacement.<br>COOT (v0.8.8), REFMAC (in CCP4 v7.0), and PHENIX v1.14 were used for model building and refinement.<br>The SPIDER2 web server (recently updated to SPIDER3; https://sparks-lab.org/server/spider3/), PSIPRED v4.0 web server (http://bioinf.cs.ucl.ac.uk/psipred/), SWISS-MODEL web server (https://swissmodel.expasy.org), and I-TASSER web server (https://zhanglab.ccmb.med.umich.edu/I-TASSER/) were used for de novo tracing and model building of cryo-EM densities.<br>Chimera v1.13 and PyMOL v2.0 were used for visual presentation of the models and maps.<br>GraphPad Prism v8.0 and Microsoft Excel were used for statistical analysis and graph drawing.

For manuscripts utilizing custom algorithms or software that are central to the research but not yet described in published literature, software must be made available to editors/reviewers. We strongly encourage code deposition in a community repository (e.g. GitHub). See the Nature Research guidelines for submitting code & software for further information.

Data

Policy information about availability of data

All manuscripts must include a data availability statement. This statement should provide the following information, where applicable:

- Accession codes, unique identifiers, or web links for publicly available datasets
- A list of figures that have associated raw data
- A description of any restrictions on data availability

Atomic coordinates, structure factors, and EM maps have been deposited in the Protein Databank and EM Databank under the following IDs: PDB: 6UT3, 6UT4, 6UT5, 6UT6, 6UT7, 6UT8; EMD-20865, EMD-20866, EMD-20867, EMD-20868, EMD-20869, EMD-20870, EMD-20871 (see Supplementary Tables S1 and S2 for full details). Figures 2d, 4d, and S1i have associated raw data. The authors declare no competing financial interests. Correspondence and requests for materials should be addressed to J.S.C. (chappie@cornell.edu) and T.W. (twalz@rockefeller.edu).

Field-specific reporting

Please select the one below that is the best fit for your research. If you are not sure, read the appropriate sections before making your selection.

☒ Life sciences ☐ Behavioural & social sciences ☐ Ecological, evolutionary & environmental sciences

For a reference copy of the document with all sections, see nature.com/documents/nr-reporting-summary-flat.pdf

Life sciences study design

All studies must disclose on these points even when the disclosure is negative.

Sample size	The sample size is critical for the resolution of a cryo-EM map that can be achieved and was determined empirically through data processing and analysis of the individual single particles selected. Since the resolution did not improve by including more particles, the sizes of the datasets were deemed sufficient. The resolution of crystal structure depends on the intrinsic order and X-ray diffraction exhibited by individual protein crystals and the phase information that can be obtained. Hundreds of crystals were screened to optimize diffraction and maximize the anomalous signal at the selenium edge for phasing. The best datasets were used for structure determination and refinement.
Data exclusions	Refinement statistics from the Tg McrB AAA Se derivative dataset are not included in Supplementary Table S1. This data was used to generate a partial, incomplete model that was not refined fully but instead was used for molecular replacement into a higher resolution native data set where refinement was completed.
Replication	Several cryo-EM data collection sessions were performed, but only the best datasets are reported and were processed as described in Methods. Measure taken to ensure reproducibility of cryo-EM sample preparation are described in the "Statistics and Reproducibility" section of the manuscript text. Hundreds of protein crystals were similarly screened for X-ray diffraction and anomalous signal and the best datasets are reported and were used for phasing, structure determination, and refinement as described in the Methods. For GTPase assays, quantified data represent the average of three independent experiments using multiple independently purified batches of protein. This is stated in the Methods.
Randomization	A complete description of the image processing steps that were used is provided in the Methods. A random set of diffraction data is set aside during crystallographic refinement for comparison to the model and used to generate the R-free statistic. Selection of these individual reflections is carried out automatically by the software and maintained throughout the refinement procedure.
Blinding	Blinding was not relevant to this study as the results could not be influenced by subjective bias. Bias introduced during crystallographic model building is accounted for in the established statistical analysis incorporated into refinement procedures. This data is included in Supplementary Table S1.

Reporting for specific materials, systems and methods

We require information from authors about some types of materials, experimental systems and methods used in many studies. Here, indicate whether each material, system or method listed is relevant to your study. If you are not sure if a list item applies to your research, read the appropriate section before selecting a response.

Materials & experimental systems

n/a	Involved in the study	
☒	☐	Antibodies
☒	☐	Eukaryotic cell lines
☒	☐	Palaeontology
☒	☐	Animals and other organisms
☒	☐	Human research participants
☒	☐	Clinical data

Methods

n/a	Involved in the study	
☒	☐	ChIP-seq
☒	☐	Flow cytometry
☒	☐	MRI-based neuroimaging
